# Effect of Early vs Delayed Surgical Treatment on Motor Recovery in Incomplete Cervical Spinal Cord Injury With Preexisting Cervical Stenosis: A Randomized Clinical Trial

**DOI:** 10.1001/jamanetworkopen.2021.33604

**Published:** 2021-11-09

**Authors:** The OSCIS investigators, Hirotaka Chikuda, Yurie Koyama, Yoshitaka Matsubayashi, Toru Ogata, Hiroshi Ohtsu, Shurei Sugita, Masahiko Sumitani, Yuho Kadono, Toshiki Miura, Sakae Tanaka, Toru Akiyama, Kei Ando, Masato Anno, Seiichi Azuma, Kenji Endo, Toru Endo, Takayuki Fujiyoshi, Takeo Furuya, Hiroyuki Hayashi, Akiro Higashikawa, Akihiko Hiyama, Chiaki Horii, Seiji Iimoto, Yoichi Iizuka, Hisanori Ikuma, Shiro Imagama, Koichi Inokuchi, Hirokazu Inoue, Tomoo Inoue, Keisuke Ishii, Masayoshi Ishii, Takui Ito, Akira Itoi, Kohei Iwamoto, Motoki Iwasaki, Takashi Kaito, Tsuyoshi Kato, Hiroyuki Katoh, Yoshiharu Kawaguchi, Osamu Kawano, Atsushi Kimura, Kazuyoshi Kobayashi, Masao Koda, Miki Komatsu, Gentaro Kumagai, Takeshi Maeda, Takahiro Makino, Chikato Mannoji, Kazuhiro Masuda, Keisuke Masuda, Koji Matsumoto, Morio Matsumoto, Shunji Matsunaga, Yukihiro Matsuyama, Tokue Mieda, Kota Miyoshi, Joji Mochida, Hiroshi Moridaira, Hiroyuki Motegi, Yukihiro Nakagawa, Yutaka Nohara, Kazunori Oae, Shinji Ogawa, Rentaro Okazaki, Akinori Okuda, Eijiro Onishi, Atsushi Ono, Masashi Oshima, Yusuke Oshita, Kazuo Saita, Yutaka Sasao, Kimiaki Sato, Kimihiko Sawakami, Atsushi Seichi, Shoji Seki, Hideki Shigematsu, Kota Suda, Yasutaka Takagi, Masahito Takahashi, Ryosuke Takahashi, Eiji Takasawa, Shota Takenaka, Katsushi Takeshita, Yujiro Takeshita, Takamitsu Tokioka, Yasuaki Tokuhashi, Juichi Tonosu, Hiroshi Uei, Kanichiro Wada, Masahiko Watanabe, Tadashi Yahata, Kei Yamada, Taketoshi Yasuda, Keigo Yasui, Toshitaka Yoshii

**Affiliations:** 1 Department of Orthopaedic Surgery, Gunma University, Maebashi, Gunma, Japan; 2 Kitasato University School of Nursing, Sagamihara, Japan; 3 Department of Orthopaedic Surgery, The University of Tokyo, Tokyo, Japan; 4 Department of Rehabilitation Medicine, The University of Tokyo, Tokyo, Japan; 5 National Center for Global Health and Medicine, Tokyo, Japan; 6 Tokyo Metropolitan Cancer and Infectious Diseases Center Komagome Hospital, Tokyo, Japan; 7 Department of Pain and Palliative Medicine, The University of Tokyo Hospital, Tokyo, Japan; 8 Saitama Medical University, Moroyama, Japan; 9 JR Tokyo General Hospital, Tokyo, Japan; 10 Saitama Medical Center, Jichi Medical University, Saitama, Japan; 11 Nagoya University Hospital, Nagoya, Japan; 12 Tokyo Metropolitan Bokutoh Hospital, Tokyo, Japan; 13 Japanese Red Cross Saitama Hospital, Saitama, Japan; 14 Tokyo Medical University, Tokyo, Japan; 15 Wakayama Medical University Hospital, Wakayama, Japan; 16 Kimitsu Chuo Hospital, Kisarazu, Japan; 17 Chiba University Hospital, Chiba, Japan; 18 Tonami General Hospital, Tonami, Japan; 19 Kanto Rosai Hospital, Kawasaki, Japan; 20 Tokai University Hospital, Isehara, Japan; 21 Hokkaido Spinal Cord Injury Center, Bibai, Japan; 22 Kagawa Prefectural Central Hospital, Takamatsu, Japan; 23 Saitama Medical University Saitama Medical Center, Kawagoe, Japan; 24 Jichi Medical University Hospital, Shimotsuke, Tochigi, Japan; 25 Kansai Rosai Hospital, Amagasaki, Japan; 26 Niigata City General Hospital, Niigata, Japan; 27 Juntendo University Shizuoka Hospital, Izunokuni, Japan; 28 Osaka Rosai Hospital, Sakai, Japan; 29 Osaka University Hospital, Suita, Japan; 30 Tokyo Medical and Dental University Hospital, Tokyo, Japan; 31 Toyama University Hospital, Toyama, Japan; 32 Spinal Injuries Center, Iizuka, Japan; 33 Hirosaki University Hospital, Hirosaki, Japan; 34 Chiba Aoba Municipal Hospital, Chiba, Japan; 35 Tokyo Metropolitan Tama Medical Center, Tokyo, Japan; 36 Nara Medical University Hospital, Kashihara, Japan; 37 Nihon University Itabashi Hospital, Tokyo, Japan; 38 Keio University Hospital, Tokyo, Japan; 39 Imakiire General Hospital, Kagoshima, Japan; 40 Hamamatsu University Hospital, Hamamatsu, Japan; 41 Yokohama Rosai Hospital, Yokohama, Japan; 42 Dokkyo Medical University Hospital, Mibu, Japan; 43 Sendai Medical Center, Sendai, Japan; 44 Kurashiki Central Hospital, Kurashiki, Okayama, Japan; 45 Showa University Northern Yokohama Hospital, Yokohama, Japan; 46 St Marianna University Hospital, Kawasaki, Japan; 47 Kurume University Hospital, Kurume, Japan; 48 Department of Orthopaedic Surgery, The University of Tokyo, Tokyo, Japan; 49 Kyorin University Hospital, Tokyo, Japan; 50 Kochi Health Sciences Center, Kochi, Japan

## Abstract

**Importance:**

The optimal management for acute traumatic cervical spinal cord injury (SCI) is unknown.

**Objective:**

To determine whether early surgical decompression results in better motor recovery than delayed surgical treatment in patients with acute traumatic incomplete cervical SCI associated with preexisting canal stenosis but without bone injury.

**Design, Setting, and Participants:**

This multicenter randomized clinical trial was conducted in 43 tertiary referral centers in Japan from December 2011 through November 2019. Patients aged 20 to 79 years with motor-incomplete cervical SCI with preexisting canal stenosis (American Spinal Injury Association [ASIA] Impairment Scale C; without fracture or dislocation) were included. Data were analyzed from September to November 2020.

**Interventions:**

Patients were randomized to undergo surgical treatment within 24 hours after admission or delayed surgical treatment after at least 2 weeks of conservative treatment.

**Main Outcomes and Measures:**

The primary end points were improvement in the mean ASIA motor score, total score of the spinal cord independence measure, and the proportion of patients able to walk independently at 1 year after injury.

**Results:**

Among 72 randomized patients, 70 patients (mean [SD] age, 65.1 [9.4] years; age range, 41-79 years; 5 [7%] women and 65 [93%] men) were included in the full analysis population (37 patients assigned to early surgical treatment and 33 patients assigned to delayed surgical treatment). Of these, 56 patients (80%) had data available for at least 1 primary outcome at 1 year. There was no significant difference among primary end points for the early surgical treatment group compared with the delayed surgical treatment group (mean [SD] change in ASIA motor score, 53.7 [14.7] vs 48.5 [19.1]; difference, 5.2; 95% CI, −4.2 to 14.5; *P* = .27; mean [SD] SCIM total score, 77.9 [22.7] vs 71.3 [27.3]; *P* = .34; able to walk independently, 21 of 30 patients [70.0%] vs 16 of 26 patients [61.5%]; *P* = .51). A mixed-design analysis of variance revealed a significant difference in the mean change in ASIA motor scores between the groups (*F*_1,49_ = 4.80; *P* = .03). The early surgical treatment group, compared with the delayed surgical treatment group, had greater motor scores than the delayed surgical treatment group at 2 weeks (mean [SD] score, 34.2 [18.8] vs 18.9 [20.9]), 3 months (mean [SD] score, 49.1 [15.1] vs 37.2 [20.9]), and 6 months (mean [SD] score, 51.5 [13.9] vs 41.3 [23.4]) after injury. Adverse events were common in both groups (eg, worsening of paralysis, 6 patients vs 6 patients; death, 3 patients vs 3 patients).

**Conclusions and Relevance:**

These findings suggest that among patients with cervical SCI, early surgical treatment produced similar motor regain at 1 year after injury as delayed surgical treatment but showed accelerated recovery within the first 6 months. These exploratory results suggest that early surgical treatment leads to faster neurological recovery, which requires further validation.

**Trial Registration:**

ClinicalTrials.gov Identifier: NCT01485458; umin.ac.jp/ctr Identifier: UMIN000006780

## Key Points

**Question:**

Does early surgical treatment yield better motor recovery than delayed surgical treatment for patients with preexisting cervical canal stenosis sustaining acute traumatic spinal cord injury?

**Findings:**

In this randomized clinical trial that included 72 patients with motor-incomplete cervical spinal cord injury, there was no statistically significant difference between groups in mean improvement in American Spinal Injury Association motor score, total score in the spinal cord independence measure, or patients’ ability to walk at 1 year. Early surgical treatment was associated with higher motor scores than delayed surgical treatment in the first 6 months.

**Meaning:**

These findings suggest that among patients with motor-incomplete cervical spinal cord injury, early surgical treatment did not significantly improve motor recovery at 1 year compared with delayed surgical treatment but showed accelerated recovery.

## Teaser

This randomized clinical trial assesses motor recovery outcomes in early vs delayed surgical treatment for patients with acute traumatic incomplete cervical spinal cord injury associated with preexisting canal stenosis.

## Introduction

Acute traumatic spinal cord injury (SCI) is a devastating condition that results in lifelong disability, long-term risks of medical complications, and use of health care resources.^1^ It is estimated that 18 000 new SCI cases occur annually in the United States.^2^ Over the past decades, the number of cervical SCIs associated with falls among the older adults has continued to increase, emerging as a major public health issue.^3,4,5^ Such SCIs are mostly incomplete, caused by low-energy trauma, and associated with preexisting canal stenosis due to degenerative changes or ossification of the posterior longitudinal ligament (OPLL).^6^

The timing of surgical treatment for SCI has long been an issue of intense debate. Although accumulated information from both experimental and clinical studies has exhibited the benefits of early surgical decompression,^7,8^ high-quality evidence to settle the argument is still limited. The superiority of early surgical decompression, especially for incomplete cervical SCI without concomitant bone injuries, has not been established, resulting in a substantial divergence in treatment choices among physicians.^9,10,11^

To address this issue, we conducted a randomized clinical trial to examine the effectiveness of early surgical treatment in patients with motor-incomplete tetraplegia associated with preexisting canal stenosis but without bone injury. The aim of this study was to determine whether early surgical decompression within 24 hours results in better motor recovery than delayed surgical treatment after at least 2 weeks of conservative treatment.

## Methods

The protocol for this randomized clinical trial in Supplement 1 was approved by the institutional review board at each participating center and was published previously.^12^ All participants provided written informed consent for the study. We followed the Consolidated Standards of Reporting Trials (CONSORT) reporting guideline.

### Study Design

The Optimal Treatment for Spinal Cord Injury associated with Cervical Canal Stenosis (OSCIS) study was a multicenter, open-label, parallel-group randomized clinical trial conducted at 43 hospitals in Japan (Trial Protocol in Supplement 1). The trial was designed according to the International Campaign for Cures of Spinal Cord Injury Paralysis guidelines.^13,14,15^

Enrollment was initiated on December 1, 2011, and closed early on November 30, 2018, owing to slow enrollment. The final follow-up of the last patient was completed in November 2019. No interim analysis was performed throughout the trial.

### Participants

All patients with acute traumatic cervical SCI who were admitted to participating hospitals within 48 hours after injury were assessed by a trial spine specialist for eligibility. Baseline neurological evaluation and imaging studies were performed prior to randomization. Patients were eligible if they were aged 20 to 79 years, with acute traumatic cervical SCI graded as American Spinal Injury Association (ASIA) Impairment Scale [AIS] C, with cervical canal stenosis owing to preexisting conditions, and without bone injury or spinal instability requiring surgical treatment. The presence and degree of canal stenosis were judged based on baseline imaging studies, including computed tomography and magnetic resonance imaging. We excluded patients if they had an unstable medical status, were unable to undergo surgical treatment within 24 hours after admission, had impaired consciousness or mental disorders that precluded neurological examination, or had difficulty granting informed consent (eTable 1 in Supplement 2).

### Randomization and Masking

Surgeons first performed baseline assessments, including neurological examination, and confirmed the eligibility of participants. After participants provided their consent and baseline information, they were randomly assigned (1:1) to receive either early surgical treatment within 24 hours after admission or delayed surgical treatment after at least 2 weeks of conservative treatment (eFigure 1 in Supplement 2). A computer-generated randomization sequence was prepared by the study statistician using blocks of 2, stratified according to high-dose methylprednisolone treatment, the presence of OPLL, preexisting gait disturbance owing to myelopathy, and severe canal compromise (>50%).^6^

Masking of trial participants and surgeons was not possible. To minimize bias in assessment, physicians and research nurses who were not involved in the patient’s care assessed the outcome at each visit before the patients met their physicians.

### Interventions

Surgical decompression was performed by or under the supervision of board-certified spine specialists. The surgical approach and spinal instrumentation were left to the surgeon’s discretion. The same surgical team performed the surgical treatment for both treatment groups. Adequate decompression was confirmed intraoperatively via direct inspection.

Patients assigned to early surgical treatment entered operation theater at the earliest opportunity and underwent surgical treatment within 24 hours after admission. Patients assigned to delayed surgical treatment first received standard conservative treatment, including intensive rehabilitation for at least 2 weeks. For safety reasons, surgeons could perform surgical decompression during the first 2 weeks in cases of neurological deterioration. If patients had satisfactory neurological recovery after 2 weeks and did not want further intervention, their surgical treatment was cancelled after consultation with physicians, as stipulated in the protocol. Otherwise, patients underwent surgical decompression, as in the early surgical treatment group, more than 2 weeks after the injury.

Both patient groups received appropriate medical treatment, including permissive or induced hypertensive therapy (mean blood pressure, >85 mm Hg) and intensive rehabilitation tailored to the individual. High-dose methylprednisolone was administered at the discretion of the treatment team following the National Acute Spinal Cord Injury Study 2 protocol.

Patients were evaluated for neurological status, including ASIA motor score on admission (before surgical treatment) and at 2 weeks, 3 months, 6 months, and 1 year after injury. We measured the spinal cord independence measure (SCIM) version 3 at 2 weeks, 3 months, 6 months, and 1 year after injury. The proportion of ambulatory patients was assessed at 1 year after injury. The Medical Outcomes Study Short Form 36 (SF-36), European Quality of Life–5 Dimension (EQ-5D), Neuropathic Pain Symptom Inventory (NPSI), and Walking Index for Spinal Cord Injury (WISCI) II were assessed at 2 weeks and 1 year after injury. Any adverse events were recorded using a web-based, predefined form. The occurrence of prespecified adverse events was assessed at each follow-up visit. Relevant information was gathered from the patients and their medical records.

### Outcomes

The primary outcome was the recovery of motor function at 1 year after injury. With no single established outcome measure, we adopted 3 end points as the primary outcomes in this exploratory trial: (1) changes from the baseline values of the ASIA motor score at 1 year after admission (range, 0 to 100; higher score indicating better motor recovery, based on 10 pairs of key muscles, each given 5 points), (2) the total score of SCIM version 3 (range, 0 to 100; higher score indicating better activity of daily living), and (3) the proportion of patients able to walk 100 m without human assistance.

Secondary outcomes included physical and mental component summary scores of SF-36, the utility scores of EQ-5D, neuropathic pain at the injured level and below as assessed by NPSI (range, 0-100; higher score indicating more severe pain), and walking status as evaluated with WISCI II (range, 0 [unable to walk] to 20 [walking without assistance for at least 10 m]; higher score indicating better walking status).

### Statistical Analysis

For this exploratory trial, the sample size (50 patients per group) was determined primarily based on feasibility. This sample size corresponds to a difference of 12 points in the ASIA motor score improvement from baseline, with an SD of 20 (45 patients per group)^16^ and also to an improvement in the percentage of ambulatory patients at 1 year from 50% to 80% (39 patients per group). All calculations assumed 80% power at a 2-tailed significance level of .05.

**Figure 1.**
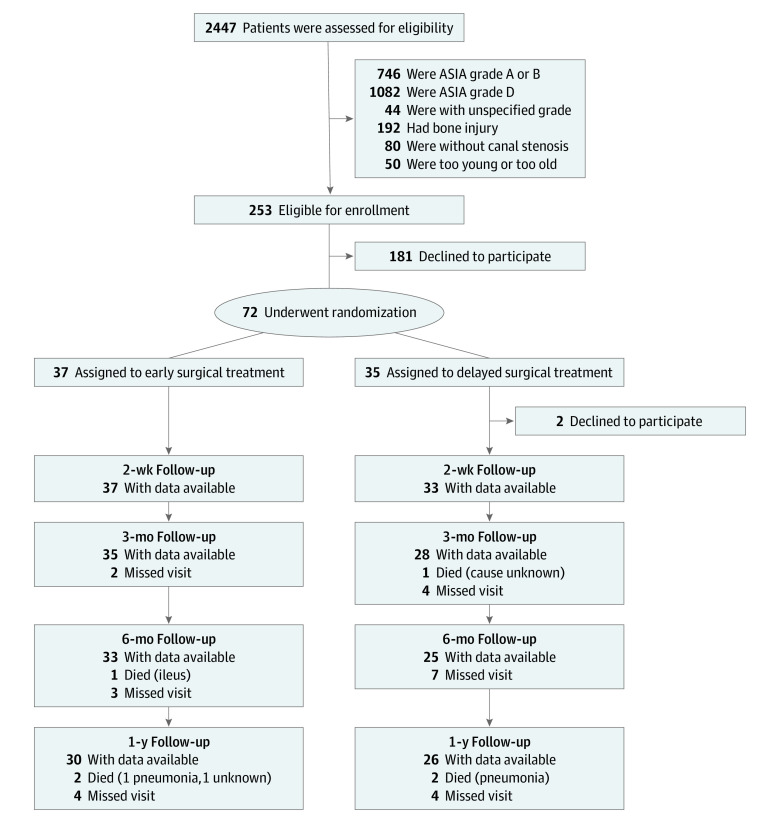
Enrollment and Randomization of Participants Patients with any of the primary end points are presented as with data available. ASIA indicates American Spinal Injury Association.

Analyses were performed on a modified intention-to-treat basis, excluding 2 patients who declined to participate immediately after randomization (Figure 1). For the ASIA motor and SCIM scores, the differences between the 2 groups were first compared using 2-way mixed-design analysis of variance as stipulated. A linear mixed-model analysis with participants as a random effect was performed. The changes from baseline in the ASIA motor scores and total score of SCIM at 1 year after admission were compared between groups using *t* test. The proportion of patients who regained walking ability was compared using the χ^2^ test. Owing to the exploratory nature of the trial, we evaluated the end points independently without adjusting multiple testing. For secondary end points, we compared the differences in SF-36, EQ-5D, NPSI, and WISCI II scores using *t* test. Adverse events were presented without statistical tests. Subgroup analyses were planned for the presence of OPLL, severe canal compromise (degree of canal compromise >50%), and central cord syndrome (defined as an upper extremity ASIA motor score of ≥10 points less than the lower extremity motor score).^17^ All analyses were performed using JMP Pro Version 14.1.0, and statistical significance was set at 2-sided *P* < .05. Data were analyzed from September to November 2020.

## Results

Among 72 randomized patients, 70 patients (mean [SD] age, 65.1 [9.4] years; age range, 41-79 years; 5 [7%] women and 65 [93%] men) were included in the full analysis population, with 37 patients randomized to early surgical treatment and 33 patients randomized to delayed surgical treatment. Two patients assigned to the delayed surgical treatment group who then declined to participate were excluded (Figure 1). Baseline patient characteristics were similar between treatment groups (Table 1). All 37 patients in the early surgical treatment group underwent surgical treatment within 24 hours after admission (median [IQR] time from admission to surgical treatment, 7.3 [4.6-12.9] hours), with 34 patients (92%) having undergone surgical treatment within 24 hours after injury. Of 33 patients assigned to the delayed surgical treatment group, 2 patients underwent surgical treatment within 2 weeks after injury, and 24 patients underwent surgical treatment after 2 weeks of conservative treatment (median [IQR] time from admission to surgical treatment, 384 [344-434] hours). The remaining 7 patients did not require surgical treatment during the study period. All surgical procedures were performed using a posterior approach (eTable 2 in Supplement 2). No patient had additional anterior surgical treatment. The follow-up rates at 1 year were similar between groups. We found no difference between the patients who were followed up and those who were lost to follow-up in baseline factors, except age (eTable 3 in Supplement 2).

**Table 1.** Baseline Characteristics of the Study Participants

Characteristic	Surgical treatment, No. (%)	
	Early (n = 37)	Delayed (n = 33)
Age, mean (SD), y	63.7 (8.9)	66.7 (9.8)
Sex		
Men	36 (97)	29 (88)
Women	1 (3)	4 (12)
Cause of injury		
Fall	27 (73)	25 (76)
Motor vehicle accident	4 (11)	4 (12)
Sports	2 (5)	1 (3)
Other	4 (11)	3 (9)
Time from injury to admission, median (IQR), min	120 (60-210)	60 (60-270)
OPLL	12 (32)	12 (36)
Degree of canal compromise >50%	11 (30)	9 (27)
Preexisting gait disturbance due to myelopathy	5 (13)	4 (12)
Motor neurologic level of injury at admission		
≤C4	15 (41)	13 (39)
C5	19 (51)	17 (52)
C6	2 (5)	2 (6)
C7	1 (3)	1 (3)
C8	0	0
T1	0	0
ASIA motor score at admission, mean (SD)		
Upper extremities	14.3 (8.7)	13.4 (8.1)
Lower extremities	19.2 (8.7)	19.4 (10.5)
Total	33.5 (10.9)	32.8 (14.0)
Central cord syndrome^a^	13 (35)	11 (33)
High-dose methylprednisolone	4 (11)	3 (9)

Abbreviations: ASIA, American Spinal Injury Association; OPLL, ossification of the posterior longitudinal ligament.

^a^ Central cord syndrome was defined as an upper extremity ASIA motor score of at least 10 points less than the lower extremity motor score.

**Figure 2.**
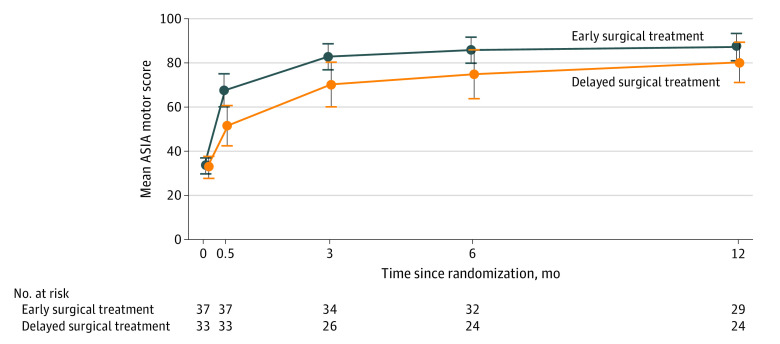
Mean ASIA Motor Score During the Study Period, According to Treatment Group ASIA indicates American Spinal Injury Association; error bars, 95% CIs.

Patients in both treatment groups showed motor recovery with major improvement during the first 6 months (Figure 2; eFigure 2 in Supplement 2). Analyses at 1 year showed a consistent effect among the primary end points, but there was no significant difference between the early surgical treatment group vs the delayed surgical treatment group (mean [SD] change ASIA motor score from baseline, 53.7 [14.7] vs 48.5 [19.1]; absolute difference, 5.2; 95% CI, −4.2 to 14.5; *P* = .27; mean [SD] SCIM total score, 77.9 [22.7] vs 71.3 [27.3]; absolute difference, 6.6; 95% CI, −7.2 to 20.4; *P* = .34; able to walk independently, 21 of 30 patients [70%] vs 16 of 26 patients [62%]; *P* = .51).

A mixed-design analysis of variance revealed a significant difference in the mean change in the ASIA motor score between the early and delayed surgical treatment groups (*F*_1,49_ = 4.80; *P* = .03; 51 patients analyzed). A linear mixed-model analysis with participants as a random effect also showed a significant effect of the intervention (ie, early surgical treatment vs delayed surgical treatment) (*F*_1,86.6_ = 11.3; *P* = .001; 70 patients analyzed). Patients in the early surgical treatment group showed better motor recovery than those in the delayed surgical treatment group at 2 weeks (mean [SD] score, 34.2 [18.8] vs 18.9 [20.9]), 3 months (mean [SD] score, 49.1 [15.1] vs 37.2 [20.9]), and 6 months (mean [SD] score, 51.5 [13.9] vs 41.3 [23.4]) after injury (Table 2). In a mixed-design analysis of variance, the difference in the total SCIM score between the groups did not reach statistical significance (eFigure 3 in Supplement 2).

**Table 2.** Mean Difference in American Spinal Injury Association Motor Score From Baseline According to Treatment

Follow-up	Early treatment		Delayed treatment		Absolute intergroup difference (95% CI)
	Motor score, mean (SD)	No.^a^	Motor score, mean (SD)	No.^a^	
2 wk	34.2 (18.8)	37	18.9 (20.9)	33	15.3 (5.9 to 24.8)
3 mo	49.1 (15.1)	34	37.2 (20.9)	26	11.9 (2.6 to 21.2)
6 mo	51.5 (13.9)	32	41.3 (23.4)	24	10.3 (0.2 to 20.3)
1 y	53.7 (14.7)	29	48.5 (19.1)	24	5.2 (−4.2 to 14.5)

^a^ Number of patients with ASIA motor score at each time point.

Patients’ quality of life assessed by SF-36 at 1 year after SCI was comparable between the early and delayed groups (mean [SD]: SF-36 physical component summary score, 13.5 [21.5] vs 11.3 [19.5]; *P* = .72; mental component summary score, 53.8 [11.8] vs 53.8 [14.4]; *P* = .99). The EQ-5D utility score continued to increase during the follow-up period in both groups (eFigure 4 in Supplement 2). Patients in the early surgical treatment group had higher EQ-5D utility scores than those in the delayed surgical treatment group, but the difference was not statistically significant (mean [SD] score, 0.739 [0.132] vs 0.695 [0.108]; absolute difference, 0.044; 95% CI, −0.026 to 0.114; *P* = .21). The early surgical treatment group had better improvement in walking status as assessed by WISCI II at 2 weeks (mean [SD], 5.7 [7.6] vs 2.3 [6.0]; *P* = .04), but the difference was not statistically significant at 1 year (mean [SD], 17.4 [5.2] vs 14.5 [7.8]; *P* = .11). The prevalence of neuropathic pain was similarly high in both groups. Early surgical treatment did not result in a reduction of neuropathic pain compared with delayed surgical treatment (mean [SD] NPSI score at 1 year: arm pain, 25.7 [23.9] vs 23.7 [20.1]; *P* = .75; trunk pain, 19.9 [22.3] vs 19.4 [19.0]; *P* = .94).

**Figure 3.**
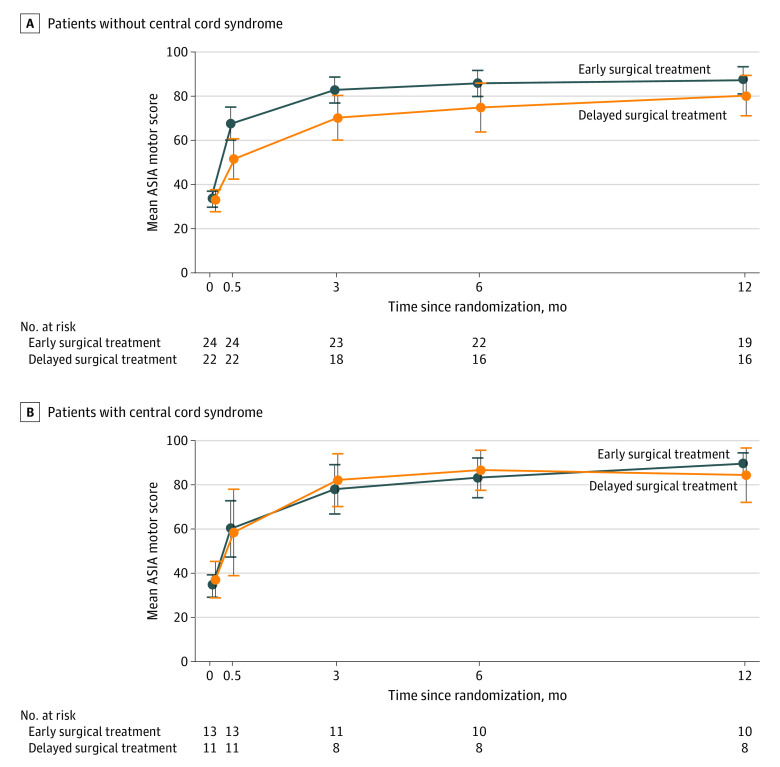
Mean ASIA Motor Score During the Study Period, Among Patients With or Without Central Cord Syndrome ASIA indicates American Spinal Injury Association; error bars, 95% CIs.

Of 70 patients, 24 (34%) met the criteria for central cord syndrome. A planned stratified analysis revealed that patients with and without central cord syndrome exhibited different responses to early surgical treatment. Enhanced recovery after early surgical treatment was observed in patients without central cord syndrome but not in those with central cord syndrome (Figure 3).

The selection of surgical procedure and surgical time were similar between the treatment groups. Patients in the early surgical treatment group were more likely to undergo surgical treatment at night and remain intubated postoperatively than those in the delayed surgical treatment group (eTable 2 in Supplement 2). Adverse events were common in both groups (eg, worsening of paralysis, 6 patients vs 6 patients; death, 3 patients vs 3 patients) (eTable 4 in Supplement 2).

## Discussion

This randomized clinical trial is the largest trial, to our knowledge, to examine the effectiveness of early surgical treatment in patients with acute traumatic cervical SCI. In this trial, we focused on incomplete cervical SCI associated with preexisting canal stenosis without bone injury. Early surgical treatment produced similar motor regain at 1 year after injury as delayed surgical treatment. Early surgical treatment was associated with greater improvement in ASIA motor scores than delayed surgical treatment at 2 weeks, 3 months, and 6 months after injury, indicating faster neurological recovery.

Neurological recovery after SCI is nonlinear; the recovery rate is rapid during the first 3 months, and motor improvement is almost complete by 9 months.^13^ In this trial, patients assigned to early surgical treatment showed greater improvement in the ASIA motor score in the first 6 months than those assigned to delayed surgical treatment. Our results suggest that early surgical treatment is associated with faster improvement compared with delayed surgical treatment. We observed a gradual decrease in intergroup differences thereafter. A plausible explanation for this finding is the ceiling effect of outcome measures adopted in our trial.^13,14^ Alternatively, this finding may be attributable to the fact that other factors, including patient age, comorbidities, and social resources, emerge as major determinants of patients’ long-term recovery.

SCIs are highly heterogeneous in terms of severity (AIS grade A-D), levels of injury, and concurrent bone injuries, which lead to variable prognoses.^15^ Recent epidemiological studies from Europe, East Asia, and North America have unanimously shown that motor-incomplete injuries (ie, AIS C and D), especially those without bone injury, are increasing, a trend possibly associated with aging of the global society.^4,5,18^ AIS C injury on admission, with a graver neurological deficit compared with AIS D, is of major clinical importance because approximately half of such patients remain nonambulatory 1 year after injury. Therefore, in this study, we focused on AIS C, in which clinical decision-making is more impending and controversial.

In this trial, we compared the 2 treatment strategies: early surgical treatment conducted within 24 hours after admission and delayed surgical treatment following at least 2 weeks of conservative treatment. We adopted a cutoff of 24 hours after admission (instead of 24 hours after injury) for safety and ethical reasons, giving patients sufficient time for consideration. An analysis of large pooled data by Badhiwala et al^19^ found that the cutoff for early surgical treatment was between 24 and 36 hours after injury. In our study, 92% of patients assigned to early surgical treatment group underwent surgical treatment 24 hours after injury. Therefore, we believe that our study is qualified to evaluate the efficacy of early surgical treatment.

With a relatively large potential for spontaneous recovery in incomplete SCI, delayed surgical treatment after 2 weeks can provide enough time for clinicians to assess the patients’ spontaneous recovery and prepare for surgical treatment. In this study, of 33 patients assigned to the delayed surgical treatment, 7 patients did not need surgical treatment during the study period. Therefore, for patients who have a high risk for urgent surgical treatment, this wait-and-see approach continues to be an option in light of comparable outcomes at 1 year between the groups.

Safety is an issue of high priority for patients with cervical SCI, as they are highly fragile and at a high risk of complications. Consistent with the results of meta-analyses,^11,20,21^ our data showed no detectable increase in the risk for complications in the early surgical treatment group, although we were unable to compare the occurrence of rare complications owing to the small number of enrolled patients. Of note, however, we found that early surgical treatment was associated with increases in night-time surgical treatment and continuing intubation after surgical treatment, which underscores the need for heightened vigilance.

Central cord syndrome, a subtype of cervical SCI characterized by greater weakness in the upper vs lower extremities, typically occurs after falls in older adults with spinal stenosis without evident fracture.^17,22,23^ Central cord syndrome is gaining attention from clinicians, with a recent increase in SCI among the older population.^24^ In this trial, we found a different response to early surgical treatment between patients with or without central cord syndrome. Enhanced recovery following early surgical treatment, as found in patients without central cord syndrome, was not observed in those with central cord syndrome. Our findings indicate that the impact of early surgical treatment differs greatly according to the characteristics and underlying pathological conditions of patients with SCI.

### Limitations

Our study has several limitations. Although OSCIS is the largest trial ever conducted to examine the effectiveness of early surgical treatment for acute traumatic cervical SCI, to our knowledge, our sample size was below the initial target. We acknowledge the potential for type 2 errors in our study. Further validation is needed for the findings of this trial. For this purpose, the minimal important difference should be established for clinical outcomes that suit best for the assessment of patients with acute traumatic SCI. Second, we also recognize that a substantial number of patients were lost to follow-up, presumably owing to the inclusion of older patients with severe trauma. However, at the final follow-up, 56 patients (80%) had data available for at least 1 primary outcome. Third, we did not standardize the rehabilitation program owing to the lack of a universally accepted treatment bundle. Still, we assume that the time and method of training did not differ greatly among patients, as they were covered by the same insurance. Fourth, we were unable to obtain detailed information regarding severity or levels of cord compression. We did not implement strict standardization of imaging protocols from a practical point of view, which precluded a detailed analysis of imaging obtained on admission. Nevertheless, owing to the nature of randomized clinical trials, we do not expect that the initial status (ie, severity) of the injured cord differs substantially between the groups. Additionally, hospitals participating in this trial were mostly large, well-staffed university hospitals. We acknowledge that there are challenges to overcome before urgent surgical treatment becomes ubiquitously available.^25^ Despite these limitations, we believe that the OSCIS study provides relevant information to help clinical decision-making.

## Conclusions

In this exploratory randomized clinical trial, early surgical decompression (<24 hours) for motor-incomplete cervical SCI without bone injury produced a comparative recovery in motor function with delayed surgical treatment (>2 weeks) at 1 year after injury. Our results suggest that early surgical treatment leads to faster neurological recovery than delayed surgical treatment, but this finding requires further validation. These findings provide crucial information for clinical decision-making, optimization of health care services, and a basis for future research.

## Notes

Supplement 1.
Trial Protocol and Statistical Analysis Plan

Supplement 2. **eTable 1.** Eligibility Criteria for Participants **eTable 2.** Intraoperative Adverse Events **eTable 3.** Baseline Characteristics of the Study Participants With or Without Primary Outcomes at 1 year **eTable 4.** Adverse Events and Complications During the Follow-up Period **eFigure 1.** Study Flowchart **eFigure 2.** Change in Motor Neurological Level of Injury During the Study Period, According to Treatment Group **eFigure 3.** Mean SCIM Score During the Study Period, According to Treatment Group **eFigure 4.** Mean EQ-5D Utility Score During the Study Period, According to Treatment Group

Supplement 3.
Data Sharing Statement
