# Error in Figure 3

**DOI:** 10.1001/jamanetworkopen.2021.43306

**Published:** 2021-12-15

In the Original Investigation titled “Effect of Early vs Delayed Surgical Treatment on Motor Recovery in Incomplete Cervical Spinal Cord Injury With Preexisting Cervical Stenosis: A Randomized Clinical Trial,”^1^ published November 9, 2021, Figure 3A was a duplicate of Figure 2. This article has been corrected.^1^
