# Supplementary material for: Effect of Early vs Delayed Surgical Treatment on Motor Recovery in Incomplete Cervical Spinal Cord Injury With Preexisting Cervical Stenosis: A Randomized Clinical Trial
Source: JAMA Netw Open. 2021 Nov 9;4(11):e2133604. doi: 10.1001/jamanetworkopen.2021.33604 (PMC8579238; doi:10.1001/jamanetworkopen.2021.33604)
Supplement: Supplement 1. — Trial Protocol and Statistical Analysis Plan [file jamanetwopen-e2133604-s001.pdf]

This supplement contains the following items:

1. Original protocol, final protocol, summary of changes.
2. Original statistical analysis plan, final statistical analysis plan, summary of changes.

Table of contents

Original Protocol version 1.1 2

Final Protocol version 1.3.3 23

Amendments to the Original Protocol 44

Original statistical analysis plan version 1.1 47

Final statistical analysis plan version 1.2 66

Amendments to the original statistical analysis plan 85

Protocol

This trial protocol has been provided by the authors to give readers additional information about their work.

Protocol for: Chikuda H, et al.,

OSCIS study: Optimal treatment for Spinal Cord Injury associated with cervical canal Stenosis Study

Study representative:
Hirotaka Chikuda

Contents

Protocol synopsis 5
1. Background 8
2. Purpose 9
3. Objectives 9
3-1. Primary objective 9
3-2. Secondary objectives 9
4. Study design 10
4-1. Overview 10
4-2. Sample size 10
4-3. Study visit 10
4-4. Study period 11
5. Population 11
5-1. Inclusion criteria 11
5-2. Exclusion criteria 12
6. Treatment 12
6-1. Treatment arms 12
6-2. Concomitant treatment 12
6-3. Treatment assignment 13
6-4. Treatment blinding 13
6-5. Study completion 13
6-6. Early study termination 13
7. Visit schedule and assessments 14
7-1. Patients demographics 14
7-2. Planned visit and assessments 14
7-3. Efficacy 15
7-4. Primary outcomes 15
7-5. Secondary outcomes 15
7-6. Safety 16
8. Safety monitoring 16
8-1. Adverse events 16
8-2. Serious adverse event reporting 16
8-3. Data monitoring committee 16
9. Termination, withdrawal, and discontinuation of research 16
9-1. Termination of research 17
9-2. Withdrawal, and discontinuation of research 17

10. Data review and database management 17
11. Data analysis 17
11-1. Population for analysis........ 17
11-2. Treatments 17
11-3. Analysis of the primary objective 17
11-4. Analysis of the secondary objectives........ 18
11-5. safety........ 18
11-6. Planned subgroup analyses 18
12. Ethical considerations 18
12-1. Ethical compliance........ 18
12-2. Informed consent procedures........ 18
13. Protocol adherence 19
14. Protocol Amendment 19
15. References 19

Protocol synopsis

Title of study

Optimal treatment for Spinal Cord Injury associated with cervical canal Stenosis Study (OSCIS Study)

Purpose

The purpose of this study is to test the hypothesis that for patients with acute cervical spinal cord injury (SCI) associated with canal stenosis, early surgery (within 24 hours after admission) will lead to better clinical outcomes compared to delayed surgery (later than two weeks after injury).

Objectives

The primary objective of this study is to test if early surgery (within 24 hours after admission) will lead to greater improvements in the motor function compared to delayed surgery (later than two weeks after injury) in patients with acute cervical SCI associated with canal stenosis.

Population

Patient who suffers acute traumatic cervical SCI and are admitted to one of the study group institutions within 48 hours after the injury.

Inclusion criteria

•acute traumatic cervical SCI (at C5 or below)
•aged 20 to 79 years
•without bone injury (spinal fracture or dislocation)
•American Spinal Injury Association (ASIA) impairment Grade C
•cervical canal stenosis due to preexisting conditions, such as spondylosis and ossification of the posterior longitudinal ligament (OPLL)

The presence of cervical canal stenosis will be confirmed by physicians based on the magnetic resonance imaging (MRI) findings obtained on admission. The presence of OPLL will be determined by using plain radiographs or computed tomography (CT). The thickness of the OPLL must be 20% or more of the spinal canal.

Exclusion criteria

•unstable medical status
•unable to undergo surgery within 24 hours after admission
•impaired consciousness or mental disorder that precludes neurological examination
•difficulty in obtaining informed consent in Japanese

Treatment

Early surgery in which patients are allocated to early surgery will undergo surgery within 24 hours after admission, or delayed surgery in which patients receive conservative treatment consisting of early mobilization and intensive rehabilitation for at least two weeks after the injury.

Study design

Randomized, controlled, parallel-group, assessor-blinded, multicenter study.

Screening and visit

Following diagnosis of cervical SCI, a study investigator (medical doctor) will assess the eligibility of the patient and obtain a written consent. Then, participants are randomly assigned to either the early surgery or delayed surgery group. After the treatments, the participants are evaluated for neurological recovery at 2 weeks, 3 months, 6 months, and 1 year after admission.

Safety

The condition of the patients will be monitored by the medical doctors recording adverse events.

Primary assessment

ASIA motor score, the proportion of patients who regained the ability to walk, and total score of Spinal Cord Independence Measure version Ⅲ（SCIM Ⅲ）.

Secondary assessment

Short-Form Health Survey-36 (SF-36), European Quality of life-5 Dimensions (EQ-5D), Neuropathic Pain Symptom Inventory (NPSI), and Walking Index for Spinal Cord Injury Ⅱ (WISCI Ⅱ)

Safety assessment

The occurrence of pre-specified adverse events (AEs) will be recorded. AEs will be gathered from patients themselves and from the patient record review. Severe AEs are defined as death, worsening of paralysis, unexpected hospital stay extension. When any of investigators recognizes severe AEs occurring on the participant, they must report to the director of their hospital or institutional review board (IRB).

Data analysis

The primary and secondary analyses will be performed in full analysis population.

For the primary objectives:

For the ASIA motor and SCIM III scores, the changes from baseline in the ASIA motor scores and total SCIM III score at one year after admission will be compared between two groups using Student's t test. The proportion of patients who regained the ability to walk will be compared using the chi-square test.

For the secondary objectives;

We will compare the differences in the SF-36, the EQ-5D, the NPSI and the WISCI II, using Student's-t test. The rates of AEs between the groups will be compared using the chi-square test or Fisher's exact test.

Planned subgroup analyses

Predefined subgroup analyses will be performed for the following factors: use of high-dose methylprednisolone treatment, the presence of OPLL, preexisting gait disturbance and severe canal compromise (> 50% canal compromise).

1. Background

Acute cervical SCI is one of the most devastating conditions, and can lead to paralysis, sensory impairment and bowel, bladder and sexual dysfunction. In addition, patients frequently suffer from intractable pain caused by neural damage. Individuals with cervical canal stenosis are known to develop cervical SCI even after minor trauma. Cervical canal stenosis may be congenital, but often results from degenerative conditions, such as spondylosis. The SCI patients with canal stenosis are mostly elderly, and usually present with incomplete SCI without bone injury, such as spinal fracture or dislocation. This subgroup of patients has been steadily increasing as the society ages and currently accounts for over 60% of cervical SCIs in Japan.

The clinical outcome of patients with incomplete SCI has been considered to be favorable, since patients usually show spontaneous neurologic recovery to some extent. However, the neurological prognosis varies greatly among patients; about half of ASIA C patients remain non-ambulatory six months after the injury [2]. In particular, the clinical outcomes of elderly patients are often suboptimal [3,4]. Therefore, a therapeutic option that leads to a better clinical outcome is urgently needed.

Controversy exists with regard to the efficacy of surgical decompression in the treatment of cervical SCI with preexisting canal stenosis [5,6]. The role of surgery remains unclear, especially in the absence of instability of the cervical spine [7], thus resulting in a significant difference in practice between institutions. A common approach to treating these patients has been to rule out acute instability and then observe the patients' spontaneous neurological recovery until they achieve a neurological plateau, and only then consider the possibility of surgical decompression, weeks after the initial injury [6].

The main drawback of this 'watch and wait' strategy is that a potential therapeutic window in the acute phase might be missed. The current concept of the pathophysiology of SCI classifies the spinal damage into two stages: primary injury and secondary injury [9]. The primary injury results from the mechanical forces delivered to the spinal cord at the time of the trauma. Secondary injury is a cascade of pathophysiological events including edema, ischemia, inflammation and apoptosis following the initial impact, which develops within minutes to hours following the trauma. There is a growing body of evidence from preclinical or animal studies that early surgical decompression alleviates 'secondary injury' and thus results in enhanced neurological and functional recovery [5].

Although numerous studies have been performed to examine the potential benefit of early surgery, the results of these prior clinical studies were mixed, and failed to provide robust support for the hypothesis that early surgery leads to improved outcomes. One small randomized trial of 42 patients showed no benefit to early (< 72 hours) decompression [10]. On the other hand, a meta-analysis of case series showed that early (< 24 hours) decompression was associated with better outcomes compared to both delayed (> 24 hours) and conservative treatment [11].

With such conflicting information in the literature and a lack of high-quality evidence, it remains unclear whether early surgical decompression would result in better neurological and functional recovery. To address this issue, we launched the OSCIS study (Optimal treatment for Spinal Cord Injury associated with cervical canal Stenosis), a randomized, controlled, multicenter trial, in which we will compare the two strategies: early surgery within 24 hours after admission and delayed surgery following at least two weeks of conservative treatment.

2. Purpose

The purpose of this study is to test the hypothesis that early surgery (within 24 hours after admission) will lead to better clinical outcomes compared to delayed surgery (later than two weeks after injury) in patients with acute cervical SCI associated with canal stenosis.

3. Objectives

3-1. Primary objective

The primary objective of this study is to test the hypothesis that early surgery (within 24 hours after admission) will lead to greater improvements in the motor function compared to delayed surgery (later than two weeks after injury) in patients with acute cervical SCI associated with canal stenosis.

3-2. Secondary objectives

The secondary objective of this study is to test the hypothesis that early surgery (within 24 hours after admission) will lead to better conditions for patients with acute cervical SCI associated with canal stenosis in terms of: the health-related quality of life, as measured by SF-36 and the EQ-5D; the pain symptoms, as assessed by NPSI; and the walking status, as evaluated with WISCI II compared to delayed surgery (later than 2 weeks after injury).

4. Study design

4-1. Overview

The OSCIS study is a randomized, controlled, parallel-group, assessor-blinded, multicenter study. Patients will be randomly allocated to undergo either early surgery or delayed surgery.

4-2. Sample size

For this exploratory trial, the sample size was determined primarily based on feasibility. We assumed that it is feasible to enroll approximately 100 patients (50 patients per group) during the planned study period. As there is no valid data to indicate the optimal endpoint to evaluate the neurological and functional recovery of SCI patients, we selected three candidate endpoints as the primary endpoint: 1) the change from the baseline to one year after the admission in the ASIA motor score; 2) the proportion of patients who regained the ability to walk 100 meters without human assistance and 3) the total score of the SCIM III.

We need 45 patients per group when the difference to be detected in the ASIA motor score between the groups is 12 points and the common standard deviation is 20. Additionally, we expect that the percentage of ambulatory patients one year after the injury will increase from 50% to 80%. To detect this difference, we need 39 patients for each group. With regard to the SCIM III, there are few data that can be used as a basis for sample size calculation. For the reasons above, we set the sample size to be 50 patients per group. All calculations assume an 80% power at a two-tailed significance level of 0.05.

4-3. Study visit

Informed consent

After the admission and diagnosis of cervical SCI, a medical doctor assigned to the study visits the patients. The doctor explains to the patients about the participation in the study using a written document. Consent must be obtained by the free will of the patients.

Confirmation eligibility

The doctor obtains the demographic data and clinical information about the participants. Based on the inclusion and exclusion criteria, the doctor determines whether the participant is eligible for the study.

Assignment to treatments

After randomization, participants are assigned to either the early surgery or delayed surgery

group.

Planned visit for evaluation

The participants have visits by the physicians for evaluation at 2 weeks, 3 months, 6 months, and 1 year after admission. Participants who have missed a scheduled follow-up visit will be contacted by a study investigator at each institution. Outcome questionnaires will be collected via mail or telephone interview if rescheduling of the visit is not possible.

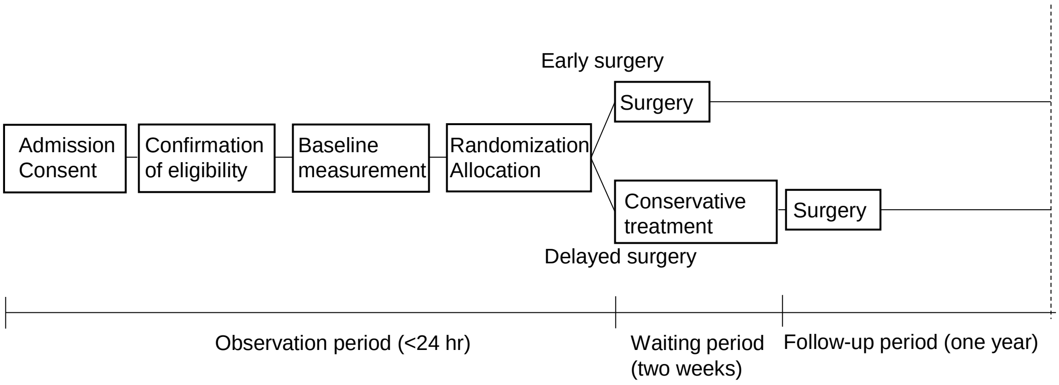

4-4. Study period

Study period: December 1, 2011 to November 30, 2013

5. Population

Subjects will be recruited from 20 hospitals in Japan. We will screen all patients with acute traumatic cervical SCI who are admitted to one of the institutions within 48 hours after the injury. The diagnosis of cervical SCI will be made on the patient's history, including physical and neurological examinations, and the results of imaging studies, including plain radiographs, MRI and CT.

5-1. Inclusion criteria

Subjects will be eligible for inclusion if they satisfy the following inclusion criteria:

•acute traumatic cervical SCI (at C5 or below)

•aged 20 to 79 years

•without bone injury (spinal fracture or dislocation)

•American Spinal Injury Association (ASIA) impairment Grade C

•cervical canal stenosis due to preexisting conditions, such as spondylosis and OPLL

The presence of cervical canal stenosis will be confirmed by physicians based on the MRI

findings obtained on admission. The presence of OPLL will be determined by using plain radiographs or CT. The thickness of the OPLL must be 20% or more of the spinal canal.

5-2. Exclusion criteria

Subjects will be excluded from enrollment if they meet any of the following conditions:
•unstable medical status
•unable to undergo surgery within 24 hours after admission
•impaired consciousness or mental disorder that precludes neurological examination
•difficulty in obtaining informed consent in Japanese

6. Treatment

6-1. Treatment arms

Patients will be randomly allocated to undergo either early surgery or delayed surgery.

Early surgery

Patients allocated to early surgery will undergo surgery within 24 hours after admission. The time when they enter the operating room will be used as a reference. The principal goal of surgery is to achieve decompression of the spinal cord. The choice of anterior or posterior approach will be left to the surgeon's discretion. The use of spinal instrumentation will be permitted when needed. The surgery will be performed by or under supervision of a board-certified orthopedic surgeon. The details of the surgical treatment and any perioperative adverse events will be recorded in a web-based predefined form. All patients will receive intensive rehabilitation tailored to the individual and injury-specific factors immediately after surgery.

Delayed surgery

Patients allocated to the delayed surgery group will receive conservative treatment consisting of early mobilization and intensive rehabilitation for at least two weeks after the injury. Surgical decompression will be performed by the same team as in the early surgery group at any time later than two weeks after the injury when the physician thinks the timing is appropriate.

6-2. Concomitant treatment

Apart from the surgical management, all patients will receive appropriate medical support, including permissive or induced hypertensive therapy (mean blood pressure > 85 mmHg) [13]. High-dose methylprednisolone will be used per the discretion of the treatment team

according to the NASCIS-2 protocol [14,15]. The use or lack of high-dose methylprednisolone must be determined and entered into the web-based database prior to the randomization. Physicians will not be allowed to change or discontinue the administration of methylprednisolone after randomization.

6-3. Treatment assignment

We will adopt the web-based allocation system using the University Medical Information Network (UMIN), which is one of the data centers that run as a public institution in Japan. By entering the information about the patient, investigators will be able to know the allocation results immediately.

The allocation table using stratified block randomization will be registered in the UMIN. The block size is concealed to all investigators involved in this study. We will adopt stratification factors as follows:

•the presence of ossification of the OPLL (yes/no)
•implementation of high-dose methylprednisolone treatment according to the NASCIS2 protocol (yes/no)
•preexisting gait disturbance due to myelopathy
•degree of canal compromise (50% or more/less than 50% canal compromise)
Preexisting gait disturbance due to myelopathy will be determined by the attending spine surgeon before randomization, based on thorough patients' history and available medical record. Gait disturbance attributable to other causes (for example, trauma, osteoarthritis, and paralysis after stroke) will be excluded.

6-4. Treatment blinding

The participants are not blinded to their treatment.
Physicians and research nurses who are not involved in the patient's care will assess the outcome at each visit before seeing their doctors.

6-5. Study completion

The final analysis will be performed when the last patient has completed 1-year follow-up or dropped out prior to the 1-year follow-up.

6-6. Early study termination

The study can be terminated at any time for any reason listed below:
•When participants decline to continue to participate.
•When participants are found to be ineligible for the study after enrollment.

•When the study is aborted.

•When the clinical doctors in charge of the participants declare the termination of enrollment.

7. Visit schedule and assessments

7-1. Patients demographics

Age—yr

Male sex—no.(%)

Etiology—no.(%) ; Fall, Motor vehicle accident, Sports, Other

Time from injury to admission—median (interquartile range) (min)

OPLL—no.(%)

Occupancy rate > 50%—no.(%)

Preexisting gait disturbance due to myelopathy—no.(%)

Motor neurologic level of injury at admission—no.(%);

7-2. Planned visit and assessments

	Admission	2 week follow-up	3 month follow-up	6 month follow-up	1 year follow-up
Target day of visit		14	90	180	365
protocol assessment time windows (days)		± 3	± 14	± 14	± 14
Visit and examination	x	x	x	x	x
Baseline clinical characteristics	x				
Blood analyses	x	x	x	x	x
Magnetic resonance imaging	x				x
Computed tomography	x				
Plain radiographs	x	x			x
Neurological assessment	x	x	x	x	x

including the ASIA motor score and ASIA impairment scale					
SCIM III	x	x	x	x	x
SF-36	x				x
EQ-5D	x	x	x	x	x
NPSI	x				x
WISCI II	x				x

7-3. Efficacy

Participants will be evaluated at 2 weeks, 3 months, 6 months and 1 year after admission. The table in 7-2 provides an overview of the outcomes that will be used in this study. Physicians and research nurses who are not involved in the patient's care will assess the outcome at each follow-up examination before the patients see their doctors.

7-4. Primary outcomes

The primary outcome is a recovery in motor function one year after injury. The assessment will include: 1) the change from baseline to one year after the admission in the ASIA motor score; 2) the total score of the SCIM III and 3) the proportion of patients who regained the ability to walk 100 meters without human assistance.

The ASIA motor score is a 100-point score based on ten pairs of key muscles, each given a five point rating. The SCIM III is a validated 100-point disability scale developed specifically for patients with SCI, with an emphasis on daily tasks grouped into three subscales: self-care (20 points), respiration and sphincter management (40 points) and mobility (40 points) [16-18].

7-5. Secondary outcomes

The secondary outcomes will include: 1) the health-related quality of life as measured by the SF-36 [19,20] and the EQ-5D [21]; 2) the neuropathic pain at the injured level and below as assessed by the NPSI [22] and 3) the walking status as evaluated with the WISCI II [23].

The scores on the SF-36 will be used as a generic measure of the patient health status. The SF-36 comprises eight single subscale scores associated with physical and mental health. The NPSI is a self-questionnaire specifically designed to evaluate the different symptoms of neuropathic pain. It includes 12 items, each of which is quantified on a (0 to 10) numerical scale. The pain associated with SCI is classified into two categories: at-level pain

and below-level pain. Participants will be asked to complete the NPSI separately for pain in the upper extremities (at-level pain) and in the trunk and lower extremities (below-level pain). The WISCI II is a valid 21-level hierarchical scale of walking based on physical assistance, the need for braces and devices, with an ordinal range from 0 (unable to walk) to 20 (walking without assistance for at least 10 meters).

7-6. Safety

The condition of the patients will be monitored by the medical doctors recording AEs. When the doctor considers that continuation is not appropriate, the follow-up of the patients will be terminated. The occurrence frequency of the adverse events will be compared between the treatment groups.

8. Safety monitoring

8-1. Adverse events

The occurrence of pre-specified AEs will be also assessed. AEs will be gathered from patients themselves and from the patient record review. The a priori defined AEs are: worsening of paralysis in the upper extremities, worsening of paralysis in the lower extremities, reoperation, use of a respirator (more than one week), tracheostomy, sepsis, pneumonia, acute respiratory distress syndrome, atelectasis, other respiratory complications, wound infection (superficial), wound infection (deep), urinary tract infection, other infections, gastrointestinal bleeding, peptic ulcer, ileus, acute myocardial infarction, other cardiac events, pulmonary embolism, cerebrovascular complication, liver dysfunction/disease, renal dysfunction/disease, delirium, depression, other complications and death.

8-2. Serious adverse event reporting

Severe AEs are defined as death, worsening of paralysis, unexpected hospital stay extension When any of investigators recognizes severe AEs occurring on the participant, they must report to the director of their hospital or IRB.

8-3. Data monitoring committee

Interim analyses are not planned.

The process of data collection and safety will be monitored by the independent safety monitoring board.

9. Termination, withdrawal, and discontinuation of

research

9-1. Termination of research

At the end of the research at each site, the investigator will submit the report of termination to the site director.

9-2. Withdrawal, and discontinuation of research

The principal investigator will consider withdrawal or discontinuation of research if any of following is applicable.

•Critical information about efficacy or safety of the protocol is obtained.

•The targeted number of enrollment seems unachievable.

•The objectives of the study are fulfilled before the planed study duration.

•IRB recommend unacceptable changes on the research protocol.

10. Data review and database management

The investigators at each site register the demographic data of participants though web-based platform to UMIN Internet Data and Information Center for Medical Research site where data will be secured.

11. Data analysis

11-1. Population for analysis

The primary and secondary analyses will be performed in full analysis population.

The full analysis population will consist of all randomized patients. The subjects who decline to participate before treatment will be excluded.

11-2. Treatments

The collected data will be analyzed according to the treatment groups, which are the early or delayed surgery groups. Analyses will be performed on an intention-to-treat basis.

11-3. Analysis of the primary objective

•ASIA motor score

The changes from baseline in the ASIA motor scores at one year after admission will be compared between two groups using Student’s t test.

•SCIM III

The total SCIM III score at one year after admission will be compared between two groups

using Student's t test.

•The proportion of patients who regained the ability to walk

The proportion of patients who regained the ability to walk will be compared using the chi-square test.

11-4. Analysis of the secondary objectives

The differences between the treatment groups in SF-36 (physical and mental component summary scores), the EQ-5D utility score, the NPSI, and the WISCI II will be compared using Student's t-test.

11-5. safety

We will compare the occurrence of AEs between the treatment groups.–

11-6. Planned subgroup analyses

Predefined subgroup analyses will be performed for the following factors: use of high-dose methylprednisolone treatment, the presence of OPLL, preexisting gait disturbance and severe canal compromise (> 50% canal compromise). Based on our previous study, we hypothesize that early surgical decompression will be beneficial in patients with preexisting gait disturbance and those with severe canal compromise.

12. Ethical considerations

12-1. Ethical compliance

The study protocol was approved by the local ethics committees of all participating hospitals and will be done in accordance with the Declaration of Helsinki. The study will be overseen by an independent safety monitoring board. All participants will give written informed consent before entry.

Ethical approval was obtained from all participating hospitals. The results will be disseminated via the usual scientific forums, including peer-reviewed publications and presentations at international conferences.

12-2. Informed consent procedures

After the admission and diagnosis of cervical SCI, a medical doctor assigned to the study visits the patients. The doctor explains to the patients about the participation in the study using the written document. Consent must be obtained by free will of the patients.

13. Protocol adherence

•The investigators must obtain approval from the principal investigator, IRB, and hospital director before modifying treatment by deviating from the protocol.

•In cases of emergency, the investigators are allowed to deviate from the protocol without approval in advance. In such cases, the investigators must report the detail of the deviation and the reason to the principal investigator and IRB, and obtain post-approval.

• Any significant deviation from the protocol must be recorded with the reason.

14. Protocol Amendment

Any change or addition to the protocol will be recorded in a written protocol amendment.

15. References

1. Pouw MH, van Middendorp JJ, van Kampen A, Curt A, van de Meent H, Hosman AJ. Diagnostic criteria of traumatic central cord syndrome. Part 3: descriptive analyses of neurological and functional outcomes in a prospective cohort of traumatic motor incomplete tetraplegics. Spinal Cord. 2011 May;49(5):614-22. Epub 2010 Dec 14.
2. Burns SP, Golding DG, Rolle WA Jr, Graziani V, Ditunno JF Jr. Recovery of ambulation in motor-incomplete tetraplegia. Arch Phys Med Rehabil. 1997;78:1169–1172.
3. Foo D. Spinal cord injury in forty-four patients with cervical spondylosis. Paraplegia. 1986;24:301–306.
4. Cadotte DW, Fehlings M. In: Controversies in spine surgery: best evidence recommendations. Vaccaro A, Eck J, editor. New York: Thiema; 2010. Role of early decompression for spinal cord injury; pp. 86–92.
5. Lenehan B, Fisher CG, Vaccaro A, Fehlings M, Aarabi B, Dvorak MF. The urgency of surgical decompression in acute central cord injuries with spondylosis and without instability. Spine (Phila Pa 1976) 2010;35:S180–186.
6. Kawano O, Ueta T, Shiba K, Iwamoto Y. Outcome of decompression surgery for cervical spinal cord injury without bone and disc injury in patients with spinal cord compression: a multicenter prospective study. Spinal Cord. 2010;48:548–553.
7. Steinmets MP, Anderson PA, Patel R, Resnick DK. In: Atlas of spine trauma: adult and pediatric. Kim DH, Ludwig SC, Vaccaro A, Chang J, editor. Philadelphia, Pennsylvania: Saunders; 2008. Anatomy and pathophysiology of spinal cord injury; pp. 11–20.

8. Vaccaro AR, Daugherty RJ, Sheehan TP, Dante SJ, Cotler JM, Balderston RA, Herbison GJ, Northrup BE. Neurologic outcome of early versus late surgery for cervical spinal cord injury. Spine (Phila Pa 1976) 1997;22:2609–2613.
9. La Rosa G, Conti A, Cardali S, Cacciola F, Tomasello F. Does early decompression improve neurological outcome of spinal cord injured patients? Appraisal of the literature using a meta-analytical approach. Spinal Cord. 2004;42:503–512.
10. Vale FL, Burns J, Jackson AB, Hadley MN. Combined medical and surgical treatment after acute spinal cord injury: results of a prospective pilot study to assess the merits of aggressive medical resuscitation and blood pressure management. J Neurosurg. 1997;87:239–246.
11. Bracken MB, Shepard MJ, Collins WF, Holford TR, Young W, Baskin DS, Eisenberg HM, Flamm E, Leo-Summers L, Maroon J. et al. A randomized, controlled trial of methylprednisolone or naloxone in the treatment of acute spinal-cord injury. Results of the Second National Acute Spinal Cord Injury Study. N Engl J Med. 1990;322:1405–1411.
12. Ito Y, Sugimoto Y, Tomioka M, Kai N, Tanaka M. Does high dose methylprednisolone sodium succinate really improve neurological status in patient with acute cervical cord injury?: a prospective study about neurological recovery and early complications. Spine (Phila Pa 1976) 2009;34:2121–2124.
13. Bluvshtein V, Front L, Itzkovich M, Aidinoff E, Gelernter I, Hart J, Biering-Soerensen F, Weeks C, Laramee MT, Craven C, Hitzig SL, Glaser E, Zeilig G, Aito S, Scivoletto G, Mecci M, Chadwick RJ, El Masry WS, Osman A, Glass CA, Silva P, Soni BM, Gardner BP, Savic G, Bergström EM, Catz A. SCIM III is reliable and valid in a separate analysis for traumatic spinal cord lesions. Spinal Cord. 2011 Feb;49(2):292-296. Epub 2010 Sep 7.
14. Catz A, Itzkovich M, Agranov E, Ring H, Tamir A. SCIM--spinal cord independence measure: a new disability scale for patients with spinal cord lesions. Spinal Cord. 1997 Dec;35(12):850-856.
15. Itzkovich M, Gelernter I, Biering-Sorensen F, Weeks C, Laramee MT, Craven BC, Tonack M, Hitzig SL, Glaser E, Zeilig G, Aito S, Scivoletto G, Mecci M, Chadwick RJ, El Masry WS, Osman A, Glass CA, Silva P, Soni BM, Gardner BP, Savic G, Bergström EM, Bluvshtein V, Ronen J, Catz A. The Spinal Cord Independence Measure (SCIM) version III: reliability and validity in a multi-center international study. Disabil Rehabil. 2007 Dec 30;29(24):1926-1933.
16. Fukuhara S, Bito S, Green J, Hsiao A, Kurokawa K. Translation, adaptation, and validation of the SF-36 Health Survey for use in Japan. J Clin Epidemiol. 1998

Nov;51(11):1037-1044.
17. Ware JE Jr, Sherbourne CD. The MOS 36-item short-form health survey (SF-36). I. Conceptual framework and item selection. Med Care. 1992 Jun;30(6):473-483.
18. EuroQol Group. EuroQol--a new facility for the measurement of health-related quality of life. Health Policy. 1990 Dec;16(3):199-208.
19. Bouhassira D, Attal N, Fermanian J, Alchaar H, Gautron M, Masquelier E, Rostaing S, Lanteri-Minet M, Collin E, Grisart J, Boureau F. Development and validation of the Neuropathic Pain Symptom Inventory. Pain. 2004 Apr;108(3):248-257.
20. Dittuno PL, Ditunno JF Jr. Walking index for spinal cord injury (WISCI II): scale revision. Spinal Cord. 2001 Dec;39(12):654-656. Erratum in: Spinal Cord. 2009 Apr;47(4):349. Dittuno, J F Jr [corrected to Ditunno, J F Jr].

Protocol

This trial protocol has been provided by the authors to give readers additional information about their work.

Protocol for: Chikuda H, et al.,

Ver.1.1 January 7, 2011
Ver.1.2 February 18, 2013
Ver.1.3.1 April 22, 2016
Ver.1.3.2 November 30, 2017
Ver.1.3.3 November 30, 2019

OSCIS study: Optimal treatment for Spinal Cord Injury associated with cervical canal Stenosis Study

Study representative:
Hirotaka Chikuda

Contents

Protocol synopsis 5
1. Background 8
2. Purpose 9
3. Objectives 9
3-1. Primary objective 9
3-2. Secondary objectives 9
4. Study design 10
4-1. Overview 10
4-2. Sample size 10
4-3. Study visit 10
4-4. Study period 11
5. Population 11
5-1. Inclusion criteria 11
5-2. Exclusion criteria 12
6. Treatment 12
6-1. Treatment arms 12
6-2. Concomitant treatment 12
6-3. Treatment assignment 13
6-4. Treatment blinding 13
6-5. Study completion 13
6-6. Early study termination 13
7. Visit schedule and assessments 14
7-1. Patients demographics 14
7-2. Planned visit and assessments 14
7-3. Efficacy 15
7-4. Primary outcomes 15
7-5. Secondary outcomes 15
7-6. Safety 16
8. Safety monitoring 16
8-1. Adverse events 16
8-2. Serious adverse event reporting 16
8-3. Data monitoring committee 16
9. Termination, withdrawal, and discontinuation of research 17
9-1. Termination of research 17
9-2. Withdrawal, and discontinuation of research 17

10. Data review and database management 17
11. Data analysis 17
11-1. Population for analysis........ 17
11-2. Treatments 17
11-3. Analysis of the primary objective 17
11-4. Analysis of the secondary objectives........ 18
11-5. safety........ 18
11-6. Planned subgroup analyses 18
12. Ethical considerations 18
12-1. Ethical compliance........ 18
12-2. Informed consent procedures........ 19
13. Protocol adherence 19
14. Protocol Amendment 19
15. References 19

Protocol synopsis

Title of study

Optimal treatment for Spinal Cord Injury associated with cervical canal Stenosis Study (OSCIS Study)

Purpose

The purpose of this study is to test the hypothesis that for patients with acute cervical spinal cord injury (SCI) associated with canal stenosis, early surgery (within 24 hours after admission) will lead to better clinical outcomes compared to delayed surgery (later than two weeks after injury).

Objectives

The primary objective of this study is to test if early surgery (within 24 hours after admission) will lead to greater improvements in the motor function compared to delayed surgery (later than two weeks after injury) in patients with acute cervical SCI associated with canal stenosis.

Population

Patient who suffers acute traumatic cervical SCI and are admitted to one of the study group institutions within 48 hours after the injury.

Inclusion criteria

•acute traumatic cervical SCI
•aged 20 to 79 years
•without bone injury (spinal fracture or dislocation)
•American Spinal Injury Association (ASIA) impairment Grade C
•cervical canal stenosis due to preexisting conditions, such as spondylosis and ossification of the posterior longitudinal ligament (OPLL)
The presence of cervical canal stenosis will be confirmed by physicians based on the magnetic resonance imaging (MRI) findings obtained on admission. The presence of OPLL will be determined by using plain radiographs or computed tomography (CT). The thickness of the OPLL must be 20% or more of the spinal canal.

Exclusion criteria

•unstable medical status
•unable to undergo surgery within 24 hours after admission
•impaired consciousness or mental disorder that precludes neurological examination
•difficulty in obtaining informed consent in Japanese

Treatment

Early surgery in which patients are allocated to early surgery will undergo surgery within 24 hours after admission, or delayed surgery in which patients receive conservative treatment consisting of early mobilization and intensive rehabilitation for at least two weeks after the injury.

Study design

Randomized, controlled, parallel-group, assessor-blinded, multicenter study.

Screening and visit

Following diagnosis of cervical SCI, a study investigator (medical doctor) will assess the eligibility of the patient and obtain a written consent. Then, participants are randomly assigned to either the early surgery or delayed surgery group. After the treatments, the participants are evaluated for neurological recovery at 2 weeks, 3 months, 6 months, and 1 year after admission .

Safety

The condition of the patients will be monitored by the medical doctors recording adverse events.

Primary assessment

ASIA motor score, the proportion of patients who regained the ability to walk, and total score of Spinal Cord Independence Measure version Ⅲ（SCIM Ⅲ）.

Secondary assessment

Short-Form Health Survey-36 (SF-36), European Quality of life-5 Dimensions (EQ-5D), Neuropathic Pain Symptom Inventory (NPSI), and Walking Index for Spinal Cord Injury Ⅱ (WISCI Ⅱ)

Safety assessment

The occurrence of pre-specified adverse events (AEs) will be recorded. AEs will be gathered from patients themselves and from the patient record review. Severe AEs are defined as death, worsening of paralysis, unexpected hospital stay extension. When any of investigators recognizes severe AEs occurring on the participant, they must report to the director of their hospital or institutional review board (IRB).

Data analysis

The primary and secondary analyses will be performed in full analysis population.

For the primary objectives:

For the ASIA motor and SCIM III scores, the differences between the treatment groups will be compared using repeated analysis of variance. At the same time, the changes from baseline in the ASIA motor scores and total SCIM III score at one year after admission will be compared between two groups using Student’s t test. The proportion of patients who regained the ability to walk will be compared using the chi-square test.

For the secondary objectives;

We will compare the differences in the SF-36, the EQ-5D, the NPSI and the WISCI II, using Student’s-t test. The rates of AEs between the groups will be compared using the chi-square test or Fisher’s exact test.

Planned subgroup analyses

Predefined subgroup analyses will be performed for the following factors: use of high-dose methylprednisolone treatment, the presence of OPLL, preexisting gait disturbance, central cord syndrome, and severe canal compromise (> 50% canal compromise).

1. Background

Acute cervical SCI is one of the most devastating conditions, and can lead to paralysis, sensory impairment and bowel, bladder and sexual dysfunction. In addition, patients frequently suffer from intractable pain caused by neural damage. Individuals with cervical canal stenosis are known to develop cervical SCI even after minor trauma. Cervical canal stenosis may be congenital, but often results from degenerative conditions, such as spondylosis. The SCI patients with canal stenosis are mostly elderly, and usually present with incomplete SCI without bone injury, such as spinal fracture or dislocation. This subgroup of patients has been steadily increasing as the society ages and currently accounts for over 60% of cervical SCIs in Japan.

The clinical outcome of patients with incomplete SCI has been considered to be favorable, since patients usually show spontaneous neurologic recovery to some extent. However, the neurological prognosis varies greatly among patients; about half of ASIA C patients remain non-ambulatory six months after the injury [2]. In particular, the clinical outcomes of elderly patients are often suboptimal [3,4]. Therefore, a therapeutic option that leads to a better clinical outcome is urgently needed.

Controversy exists with regard to the efficacy of surgical decompression in the treatment of cervical SCI with preexisting canal stenosis [5,6]. The role of surgery remains unclear, especially in the absence of instability of the cervical spine [7], thus resulting in a significant difference in practice between institutions. A common approach to treating these patients has been to rule out acute instability and then observe the patients' spontaneous neurological recovery until they achieve a neurological plateau, and only then consider the possibility of surgical decompression, weeks after the initial injury [6].

The main drawback of this 'watch and wait' strategy is that a potential therapeutic window in the acute phase might be missed. The current concept of the pathophysiology of SCI classifies the spinal damage into two stages: primary injury and secondary injury [9]. The primary injury results from the mechanical forces delivered to the spinal cord at the time of the trauma. Secondary injury is a cascade of pathophysiological events including edema, ischemia, inflammation and apoptosis following the initial impact, which develops within minutes to hours following the trauma. There is a growing body of evidence from preclinical or animal studies that early surgical decompression alleviates 'secondary injury' and thus results in enhanced neurological and functional recovery [5].

Although numerous studies have been performed to examine the potential benefit of early surgery, the results of these prior clinical studies were mixed, and failed to provide robust support for the hypothesis that early surgery leads to improved outcomes. One small randomized trial of 42 patients showed no benefit to early (< 72 hours) decompression [10]. On the other hand, a meta-analysis of case series showed that early (< 24 hours) decompression was associated with better outcomes compared to both delayed (> 24 hours) and conservative treatment [11].

With such conflicting information in the literature and a lack of high-quality evidence, it remains unclear whether early surgical decompression would result in better neurological and functional recovery. To address this issue, we launched the OSCIS study (Optimal treatment for Spinal Cord Injury associated with cervical canal Stenosis), a randomized, controlled, multicenter trial, in which we will compare the two strategies: early surgery within 24 hours after admission and delayed surgery following at least two weeks of conservative treatment.

2. Purpose

The purpose of this study is to test the hypothesis that early surgery (within 24 hours after admission) will lead to better clinical outcomes compared to delayed surgery (later than two weeks after injury) in patients with acute cervical SCI associated with canal stenosis.

3. Objectives

3-1. Primary objective

The primary objective of this study is to test the hypothesis that early surgery (within 24 hours after admission) will lead to greater improvements in the motor function compared to delayed surgery (later than two weeks after injury) in patients with acute cervical SCI associated with canal stenosis.

3-2. Secondary objectives

The secondary objective of this study is to test the hypothesis that early surgery (within 24 hours after admission) will lead to better conditions for patients with acute cervical SCI associated with canal stenosis in terms of: the health-related quality of life, as measured by SF-36 and the EQ-5D; the pain symptoms, as assessed by NPSI; and the walking status, as evaluated with WISCI II compared to delayed surgery (later than 2 weeks after injury).

4. Study design

4-1. Overview

The OSCIS study is a randomized, controlled, parallel-group, assessor-blinded, multicenter study. Patients will be randomly allocated to undergo either early surgery or delayed surgery.

4-2. Sample size

For this exploratory trial, the sample size was determined primarily based on feasibility. We assumed that it is feasible to enroll approximately 100 patients (50 patients per group) during the planned study period. As there is no valid data to indicate the optimal endpoint to evaluate the neurological and functional recovery of SCI patients, we selected three candidate endpoints as the primary endpoint: 1) the change from the baseline to one year after the admission in the ASIA motor score; 2) the proportion of patients who regained the ability to walk 100 meters without human assistance and 3) the total score of the SCIM III.

We need 45 patients per group when the difference to be detected in the ASIA motor score between the groups is 12 points and the common standard deviation is 20. Additionally, we expect that the percentage of ambulatory patients one year after the injury will increase from 50% to 80%. To detect this difference, we need 39 patients for each group. With regard to the SCIM III, there are few data that can be used as a basis for sample size calculation. For the reasons above, we set the sample size to be 50 patients per group. All calculations assume an 80% power at a two-tailed significance level of 0.05.

4-3. Study visit

Informed consent

After the admission and diagnosis of cervical SCI, a medical doctor assigned to the study visits the patients. The doctor explains to the patients about the participation in the study using a written document. Consent must be obtained by the free will of the patients.

Confirmation eligibility

The doctor obtains the demographic data and clinical information about the participants. Based on the inclusion and exclusion criteria, the doctor determines whether the participant is eligible for the study.

Assignment to treatments

After randomization, participants are assigned to either the early surgery or delayed surgery

group.

Planned visit for evaluation

The participants have visits by the physicians for evaluation at 2 weeks, 3 months, 6 months, and 1 year after admission. Participants who have missed a scheduled follow-up visit will be contacted by a study investigator at each institution. Outcome questionnaires will be collected via mail or telephone interview if rescheduling of the visit is not possible.

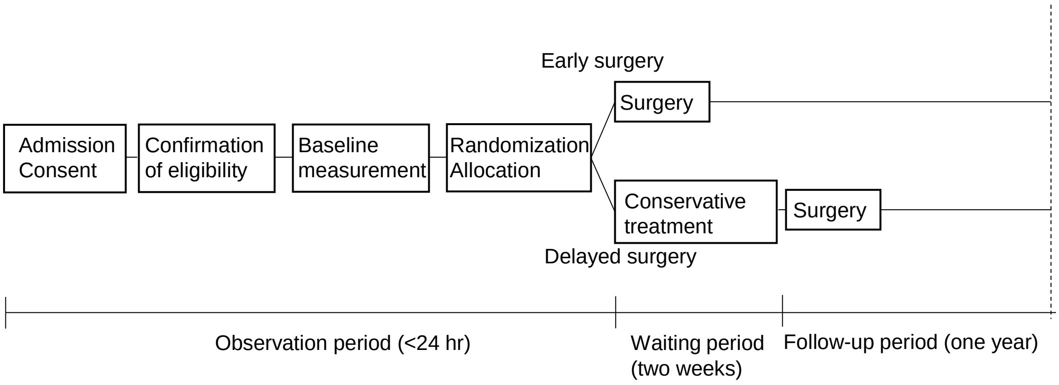

4-4. Study period

Study period: December 1, 2011 to November 30, 2020

Early termination of the trial

Due to slow enrollment, the steering committee decided the early termination of the trial in November 2017 with an intention to stop recruiting after another 1 year.

5. Population

Subjects will be recruited from 43 hospitals in Japan. We will screen all patients with acute traumatic cervical SCI who are admitted to one of the institutions within 48 hours after the injury. The diagnosis of cervical SCI will be made on the patient’s history, including physical and neurological examinations, and the results of imaging studies, including plain radiographs, MRI and CT.

5-1. Inclusion criteria

Subjects will be eligible for inclusion if they satisfy the following inclusion criteria:

•acute traumatic cervical SCI

•aged 20 to 79 years

•without bone injury (spinal fracture or dislocation)

•American Spinal Injury Association (ASIA) impairment Grade C

•cervical canal stenosis due to preexisting conditions, such as spondylosis and OPLL
The presence of cervical canal stenosis will be confirmed by physicians based on the MRI findings obtained on admission. The presence of OPLL will be determined by using plain radiographs or CT. The thickness of the OPLL must be 20% or more of the spinal canal.

5-2. Exclusion criteria

Subjects will be excluded from enrollment if they meet any of the following conditions:
•unstable medical status
•unable to undergo surgery within 24 hours after admission
•impaired consciousness or mental disorder that precludes neurological examination
•difficulty in obtaining informed consent in Japanese

6. Treatment

6-1. Treatment arms

Patients will be randomly allocated to undergo either early surgery or delayed surgery.

Early surgery

Patients allocated to early surgery will undergo surgery within 24 hours after admission. The time when they enter the operating room will be used as a reference. The principal goal of surgery is to achieve decompression of the spinal cord. The choice of anterior or posterior approach will be left to the surgeon's discretion. The use of spinal instrumentation will be permitted when needed. The surgery will be performed by or under supervision of a board-certified orthopedic surgeon. The details of the surgical treatment and any perioperative adverse events will be recorded in a web-based predefined form. All patients will receive intensive rehabilitation tailored to the individual and injury-specific factors immediately after surgery.

Delayed surgery

Patients allocated to the delayed surgery group will receive conservative treatment consisting of early mobilization and intensive rehabilitation for at least two weeks after the injury. Surgical decompression will be performed by the same team as in the early surgery group at any time later than two weeks after the injury when the physician thinks the timing is appropriate.

6-2. Concomitant treatment

Apart from the surgical management, all patients will receive appropriate medical support,

including permissive or induced hypertensive therapy (mean blood pressure > 85 mmHg) [13]. High-dose methylprednisolone will be used per the discretion of the treatment team according to the NASCIS-2 protocol [14,15]. The use or lack of high-dose methylprednisolone must be determined and entered into the web-based database prior to the randomization. Physicians will not be allowed to change or discontinue the administration of methylprednisolone after randomization.

6-3. Treatment assignment

We will adopt the web-based allocation system using the University Medical Information Network (UMIN), which is one of the data centers that run as a public institution in Japan. By entering the information about the patient, investigators will be able to know the allocation results immediately.

The allocation table using stratified block randomization will be registered in the UMIN. The block size is concealed to all investigators involved in this study. We will adopt stratification factors as follows:

•the presence of ossification of the OPLL (yes/no)
•implementation of high-dose methylprednisolone treatment according to the NASCIS2 protocol (yes/no)
•preexisting gait disturbance due to myelopathy
•degree of canal compromise (50% or more/less than 50% canal compromise)

Preexisting gait disturbance due to myelopathy will be determined by the attending spine surgeon before randomization, based on thorough patients' history and available medical record. Gait disturbance attributable to other causes (for example, trauma, osteoarthritis, and paralysis after stroke) will be excluded.

6-4. Treatment blinding

The participants are not blinded to their treatment.
Physicians and research nurses who are not involved in the patient's care will assess the outcome at each visit before seeing their doctors.

6-5. Study completion

The final analysis will be performed when the last patient has completed 1-year follow-up or dropped out prior to the 1-year follow-up.

6-6. Early study termination

The study can be terminated at any time for any reason listed below:

•When participants decline to continue to participate.
•When participants are found to be ineligible for the study after enrollment.
•When the study is aborted.
•When the clinical doctors in charge of the participants declare the termination of enrollment.

7. Visit schedule and assessments

7-1. Patients demographics

Age—yr
Male sex—no.(%)
Etiology—no.(%) ; Fall, Motor vehicle accident, Sports, Other
Time from injury to admission—median (interquartile range) (min)
OPLL—no.(%)
Occupancy rate > 50%—no.(%)
Preexisting gait disturbance due to myelopathy—no.(%)
Motor neurologic level of injury at admission—no.(%);

7-2. Planned visit and assessments

	Admission	2 week follow-up	3 month follow-up	6 month follow-up	1 year follow-up
Target day of visit		14	90	180	365
protocol assessment time windows (days)		± 3	± 14	± 14	± 14
Visit and examination	x	x	x	x	x
Baseline clinical characteristics	x				
Blood analyses	x	x	x	x	x
Magnetic resonance imaging	x				x
Computed tomography	x				
Plain radiographs	x	x			x

Neurological assessment including the ASIA motor score and ASIA impairment scale	x	x	x	x	x
SCIM III	x	x	x	x	x
SF-36	x				x
EQ-5D	x	x	x	x	x
NPSI	x				x
WISCI II	x				x

7-3. Efficacy

Participants will be evaluated at 2 weeks, 3 months, 6 months and 1 year after admission. The table in 7-2 provides an overview of the outcomes that will be used in this study. Physicians and research nurses who are not involved in the patient's care will assess the outcome at each follow-up examination before the patients see their doctors.

7-4. Primary outcomes

The primary outcome is a recovery in motor function one year after injury. The assessment will include: 1) the change from baseline to one year after the admission in the ASIA motor score; 2) the total score of the SCIM Ⅲ and 3) the proportion of patients who regained the ability to walk 100 meters without human assistance.

The ASIA motor score is a 100-point score based on ten pairs of key muscles, each given a five point rating. The SCIM III is a validated 100-point disability scale developed specifically for patients with SCI, with an emphasis on daily tasks grouped into three subscales: self-care (20 points), respiration and sphincter management (40 points) and mobility (40 points) [16-18].

7-5. Secondary outcomes

The secondary outcomes will include: 1) the health-related quality of life as measured by the SF-36 [19,20] and the EQ-5D [21]; 2) the neuropathic pain at the injured level and below as assessed by the NPSI [22] and 3) the walking status as evaluated with the WISCI II [23].

The scores on the SF-36 will be used as a generic measure of the patient health status. The SF-36 comprises eight single subscale scores associated with physical and mental health. The NPSI is a self-questionnaire specifically designed to evaluate the different

symptoms of neuropathic pain. It includes 12 items, each of which is quantified on a (0 to 10) numerical scale. The pain associated with SCI is classified into two categories: at-level pain and below-level pain. Participants will be asked to complete the NPSI separately for pain in the upper extremities (at-level pain) and in the trunk and lower extremities (below-level pain). The WISCI II is a valid 21-level hierarchical scale of walking based on physical assistance, the need for braces and devices, with an ordinal range from 0 (unable to walk) to 20 (walking without assistance for at least 10 meters).

7-6. Safety

The condition of the patients will be monitored by the medical doctors recording AEs. When the doctor considers that continuation is not appropriate, the follow-up of the patients will be terminated. The occurrence frequency of the adverse events will be compared between the treatment groups.

8. Safety monitoring

8-1. Adverse events

The occurrence of pre-specified AEs will be also assessed. AEs will be gathered from patients themselves and from the patient record review. The a priori defined AEs are: worsening of paralysis in the upper extremities, worsening of paralysis in the lower extremities, reoperation, use of a respirator (more than one week), tracheostomy, sepsis, pneumonia, acute respiratory distress syndrome, atelectasis, other respiratory complications, wound infection (superficial), wound infection (deep), urinary tract infection, other infections, gastrointestinal bleeding, peptic ulcer, ileus, acute myocardial infarction, other cardiac events, pulmonary embolism, cerebrovascular complication, liver dysfunction/disease, renal dysfunction/disease, delirium, depression, other complications and death.

8-2. Serious adverse event reporting

Severe AEs are defined as death, worsening of paralysis, unexpected hospital stay extension When any of investigators recognizes severe AEs occurring on the participant, they must report to the director of their hospital or IRB.

8-3. Data monitoring committee

Interim analyses are not planned.

The process of data collection and safety will be monitored by the independent safety monitoring board.

9. Termination, withdrawal, and discontinuation of research

9-1. Termination of research

At the end of the research at each site, the investigator will submit the report of termination to the site director.

9-2. Withdrawal, and discontinuation of research

The principal investigator will consider withdrawal or discontinuation of research if any of following is applicable.

•Critical information about efficacy or safety of the protocol is obtained.

•The targeted number of enrollment seems unachievable.

•The objectives of the study are fulfilled before the planed study duration.

•IRB recommend unacceptable changes on the research protocol.

10. Data review and database management

The investigators at each site register the demographic data of participants though web-based platform to UMIN Internet Data and Information Center for Medical Research site where data will be secured.

11. Data analysis

11-1. Population for analysis

The primary and secondary analyses will be performed in full analysis population.

The full analysis population will consist of all randomized patients. The subjects who decline to participate before treatment will be excluded.

11-2. Treatments

The collected data will be analyzed according to the treatment groups, which are the early or delayed surgery groups. Analyses will be performed on an intention-to-treat basis.

11-3. Analysis of the primary objective

•ASIA motor score

The differences between the treatment groups will be compared using repeated analysis of variance. At the same time, the changes from baseline in the ASIA motor scores at one year

after admission will be compared between two groups using Student’s t test.

•SCIM III

The differences between the treatment groups will be compared using repeated analysis of variance. At the same time, the total SCIM III score at one year after admission will be compared between two groups using Student’s t test.

•The proportion of patients who regained the ability to walk

The proportion of patients who regained the ability to walk will be compared using the chi-square test.

11-4. Analysis of the secondary objectives

The differences between the treatment groups in SF-36 (physical and mental component summary scores), the EQ-5D utility score, the NPSI, and the WISCI II will be compared using Student’s t-test.

11-5. safety

We will compare the occurrence of AEs between the treatment groups.–

11-6. Planned subgroup analyses

Predefined subgroup analyses will be performed for the following factors: use of high-dose methylprednisolone treatment, the presence of OPLL, preexisting gait disturbance, central cord syndrome, and severe canal compromise (> 50% canal compromise). Based on our previous study, we hypothesize that early surgical decompression will be beneficial in patients with preexisting gait disturbance and those with severe canal compromise.

12. Ethical considerations

12-1. Ethical compliance

The study protocol was approved by the local ethics committees of all participating hospitals and will be done in accordance with the Declaration of Helsinki. The study will be overseen by an independent safety monitoring board. All participants will give written informed consent before entry.

Ethical approval was obtained from all participating hospitals. The results will be disseminated via the usual scientific forums, including peer-reviewed publications and presentations at international conferences.

12-2. Informed consent procedures

After the admission and diagnosis of cervical SCI, a medical doctor assigned to the study visits the patients. The doctor explains to the patients about the participation in the study using the written document. Consent must be obtained by free will of the patients.

13. Protocol adherence

•The investigators must obtain approval from the principal investigator, IRB, and hospital director before modifying treatment by deviating from the protocol.
•In cases of emergency, the investigators are allowed to deviate from the protocol without approval in advance. In such cases, the investigators must report the detail of the deviation and the reason to the principal investigator and IRB, and obtain post-approval.
• Any significant deviation from the protocol must be recorded with the reason.

14. Protocol Amendment

Any change or addition to the protocol will be recorded in a written protocol amendment.

15. References

1. Pouw MH, van Middendorp JJ, van Kampen A, Curt A, van de Meent H, Hosman AJ. Diagnostic criteria of traumatic central cord syndrome. Part 3: descriptive analyses of neurological and functional outcomes in a prospective cohort of traumatic motor incomplete tetraplegics. Spinal Cord. 2011 May;49(5):614-22. Epub 2010 Dec 14.
2. Burns SP, Golding DG, Rolle WA Jr, Graziani V, Ditunno JF Jr. Recovery of ambulation in motor-incomplete tetraplegia. Arch Phys Med Rehabil. 1997;78:1169–1172.
3. Foo D. Spinal cord injury in forty-four patients with cervical spondylosis. Paraplegia. 1986;24:301–306.
4. Cadotte DW, Fehlings M. In: Controversies in spine surgery: best evidence recommendations. Vaccaro A, Eck J, editor. New York: Thiema; 2010. Role of early decompression for spinal cord injury; pp. 86–92.
5. Lenehan B, Fisher CG, Vaccaro A, Fehlings M, Aarabi B, Dvorak MF. The urgency of surgical decompression in acute central cord injuries with spondylosis and without instability. Spine (Phila Pa 1976) 2010;35:S180–186.
6. Kawano O, Ueta T, Shiba K, Iwamoto Y. Outcome of decompression surgery for cervical spinal cord injury without bone and disc injury in patients with spinal cord compression:

a multicenter prospective study. Spinal Cord. 2010;48:548–553.

7. Steinmets MP, Anderson PA, Patel R, Resnick DK. In: Atlas of spine trauma: adult and pediatric. Kim DH, Ludwig SC, Vaccaro A, Chang J, editor. Philadelphia, Pennsylvania: Saunders; 2008. Anatomy and pathophysiology of spinal cord injury; pp. 11–20.
8. Vaccaro AR, Daugherty RJ, Sheehan TP, Dante SJ, Cotler JM, Balderston RA, Herbison GJ, Northrup BE. Neurologic outcome of early versus late surgery for cervical spinal cord injury. Spine (Phila Pa 1976) 1997;22:2609–2613.
9. La Rosa G, Conti A, Cardali S, Cacciola F, Tomasello F. Does early decompression improve neurological outcome of spinal cord injured patients? Appraisal of the literature using a meta-analytical approach. Spinal Cord. 2004;42:503–512.
10. Vale FL, Burns J, Jackson AB, Hadley MN. Combined medical and surgical treatment after acute spinal cord injury: results of a prospective pilot study to assess the merits of aggressive medical resuscitation and blood pressure management. J Neurosurg. 1997;87:239–246.
11. Bracken MB, Shepard MJ, Collins WF, Holford TR, Young W, Baskin DS, Eisenberg HM, Flamm E, Leo-Summers L, Maroon J. et al. A randomized, controlled trial of methylprednisolone or naloxone in the treatment of acute spinal-cord injury. Results of the Second National Acute Spinal Cord Injury Study. N Engl J Med. 1990;322:1405–1411.
12. Ito Y, Sugimoto Y, Tomioka M, Kai N, Tanaka M. Does high dose methylprednisolone sodium succinate really improve neurological status in patient with acute cervical cord injury?: a prospective study about neurological recovery and early complications. Spine (Phila Pa 1976) 2009;34:2121–2124.
13. Bluvshtein V, Front L, Itzkovich M, Aidinoff E, Gelernter I, Hart J, Biering-Soerensen F, Weeks C, Laramee MT, Craven C, Hitzig SL, Glaser E, Zeilig G, Aito S, Scivoletto G, Mecci M, Chadwick RJ, El Masry WS, Osman A, Glass CA, Silva P, Soni BM, Gardner BP, Savic G, Bergström EM, Catz A. SCIM III is reliable and valid in a separate analysis for traumatic spinal cord lesions. Spinal Cord. 2011 Feb;49(2):292-296. Epub 2010 Sep 7.
14. Catz A, Itzkovich M, Agranov E, Ring H, Tamir A. SCIM--spinal cord independence measure: a new disability scale for patients with spinal cord lesions. Spinal Cord. 1997 Dec;35(12):850-856.
15. Itzkovich M, Gelernter I, Biering-Sorensen F, Weeks C, Laramee MT, Craven BC, Tonack M, Hitzig SL, Glaser E, Zeilig G, Aito S, Scivoletto G, Mecci M, Chadwick RJ, El Masry WS, Osman A, Glass CA, Silva P, Soni BM, Gardner BP, Savic G, Bergström EM, Bluvshtein V, Ronen J, Catz A. The Spinal Cord Independence Measure (SCIM) version

III: reliability and validity in a multi-center international study. Disabil Rehabil. 2007 Dec 30;29(24):1926-1933.
16. Fukuhara S, Bito S, Green J, Hsiao A, Kurokawa K. Translation, adaptation, and validation of the SF-36 Health Survey for use in Japan. J Clin Epidemiol. 1998 Nov;51(11):1037-1044.
17. Ware JE Jr, Sherbourne CD. The MOS 36-item short-form health survey (SF-36). I. Conceptual framework and item selection. Med Care. 1992 Jun;30(6):473-483.
18. EuroQol Group. EuroQol--a new facility for the measurement of health-related quality of life. Health Policy. 1990 Dec;16(3):199-208.
19. Bouhassira D, Attal N, Fermanian J, Alchaar H, Gautron M, Masquelier E, Rostaing S, Lanteri-Minet M, Collin E, Grisart J, Boureau F. Development and validation of the Neuropathic Pain Symptom Inventory. Pain. 2004 Apr;108(3):248-257.
20. Dittuno PL, Ditunno JF Jr. Walking index for spinal cord injury (WISCI II): scale revision. Spinal Cord. 2001 Dec;39(12):654-656. Erratum in: Spinal Cord. 2009 Apr;47(4):349. Dittuno, J F Jr [corrected to Ditunno, J F Jr].

Amendments to the Original Protocol

Ver 1.2 February 18, 2013

No.	item	Before changed	After changed	Rationale
1	4-4 Study period	December 1, 2011 to November 30, 2013	December 1, 2011 to November 30, 2017	Slow enrollment
2	5.Population	recruited from 20 hospitals	recruited from 28 hospitals	Increase of participating institutions
3	5-1. Inclusion criteria	acute traumatic cervical spinal cord injury (at C5 or below)	acute traumatic cervical spinal cord injury	ASIA Impairment Scale C patients, especially those with central cord syndrome, often presented with C4 injury.

Ver. 1.3.1 April 22, 2016

No.	item	Before changed	After changed	Rationale
1	4-4 Study period	to November 30, 2017	to November 30, 2020	Slow enrollment
2	5.Population	recruited from 28 hospitals	recruited from 42 hospitals	Increase of participating institutions

Ver. 1.3.2 November 30, 2017

No.	item	Before changed	After changed	Rationale
1	4-4. Study period Early termination of the trial	The initial protocol planned to enroll 100 patients.	Due to slow enrollment, the steering committee declared the early termination with intention to continue enrollment for another 1 year.	Slow enrollment due to other potentially competing spinal cord injury trials

2	5.Population	recruited from 42 hospitals	recruited from 43 hospitals	Increase of participating institutions

Ver. 1.3.3 November 30, 2019

No.	item	Before changed	After changed	Rationale
1	11-3 Analysis of the primary objective<br><br>ASIA motor score	The changes from baseline in the ASIA motor scores at one year after admission will be compared between two groups using Student's t test.	The differences between the treatment groups will be compared using a repeated analysis of variance. The changes from baseline in the ASIA motor scores at one year after admission will be compared between two groups using Student's t test.	Modified to fit the statistical analysis plan
2	11-3 Analysis of the primary objective<br><br>SCIM III	The total SCIM III score at one year after admission will be compared between two groups using Student's t test.	The differences between the treatment groups will be compared using repeated analysis of variance. At the same time, the total SCIM III score at one year after admission will be compared between two groups using Student's t test.	Modified to fit the statistical analysis plan

2 / 3

3	11-6 Planned subgroup analyses	Predefined subgroup analyses will be performed for the following factors: use of high-dose methylprednisolone treatment, the presence of OPLL, preexisting gait disturbance, and severe canal compromise (> 50% canal compromise).	Predefined subgroup analyses will be performed for the following factors: use of high-dose methylprednisolone treatment, the presence of OPLL, preexisting gait disturbance, central cord syndrome, and severe canal compromise (> 50% canal compromise).	Modified to fit the statistical analysis plan

Abbreviations: ASIA, American Spinal Injury Association; OPLL, ossification of the posterior longitudinal ligament; SCIM III, Spinal Cord Independence Measure version III

Statistical Analysis Plan

TRIAL FULL TITLE	Optimal treatment for Spinal Cord Injury associated with cervical canal Stenosis Study (OSCIS Study)
REGISTRATION NUMBER	NCT01485458; UMIN000006780
SAP VERSION	Ver. 1.1
SAP VERSION DATE	30/11/2018
TRIAL STATISTICIAN	Hiroshi Ohtsu
TRIAL CHIEF INVESTIGATOR	Hirotaka Chikuda
SAP AUTHOR	Hiroshi Ohtsu

1 SAP Signatures

I give my approval for the attached SAP for the randomized controlled trial entitled OSICS, dated 30/11/2018.

Chief Investigator

Name: Hirotaka Chikuda

Signature: Hirotaka Chikuda

Date: 30/11/2018

Statistician

Name: Hiroshi Ohtsu

Signature: H Ohtsu

Date: 30/11/2018

2 Table of Contents

1 SAP Signatures 1
2 Table of Contents 2
3 Abbreviations and Definitions 5
4 Introduction 6
4.1 Preface 6
4.2 Scope of the analyses 6
5 Study Objectives and Endpoints 6
5.1 Study Objectives 6
5.2 Endpoints 6
6 Study Methods 7
6.1 General Study Design and Plan 7
6.2 Equivalence or Non-Inferiority Studies 8
6.3 Inclusion-Exclusion Criteria and General Study Population 8
6.4 Randomization and Blinding 9
6.5 Study Variables 9
7 Sample Size 11
8 General Considerations 11
8.1 Timing of Analyses 11
8.2 Analysis Populations 11
8.2.1 Full Analysis Population 11
8.2.2 Per Protocol Population 11
8.2.3 Safety Population 12
8.3 Covariates and Subgroups 12
8.4 Missing Data 12
8.5 Interim Analyses and Data Monitoring 12
8.5.1 Purpose of Interim Analyses 12

8.5.2 Planned Schedule of Interim Analyses 12
8.5.3 Scope of Adaptations 13
8.5.4 Stopping Rules 13
8.5.5 Analysis Methods to Minimise Bias 13
8.5.6 Adjustment of Confidence Intervals and p-values 13
8.5.7 Interim Analysis for Sample Size Adjustment 13
8.5.8 Practical Measures to Minimise Bias 13
8.5.9 Documentation of Interim Analyses 13
8.6 Multi-centre Studies 13
8.7 Multiple Testing 13
9 Summary of Study Data 13
9.1 Subject Disposition 14
9.2 Derived variables 14
9.3 Protocol Deviations 15
9.4 Demographic and Baseline Variables 15
9.5 Concurrent Illnesses and Medical Conditions 15
9.6 Prior and Concurrent Medications 15
9.7 Treatment Compliance 15
10 Efficacy Analyses 15
10.1 Primary Efficacy Analysis 16
10.2 Secondary Efficacy Analyses 16
10.3 Exploratory Efficacy Analyses 17
11 Safety Analyses 17
11.1 Extent of Exposure 17
11.2 Adverse Events 17
11.3 Deaths, Serious Adverse Events and other Significant Adverse Events 17
11.4 Pregnancies 17
11.5 Clinical Laboratory Evaluations 18

11.6 Other Safety Measures 18
12 Pharmacokinetics 18
13 Other Analyses 18
14 Reporting Conventions 18
15 Technical Details 18
16 References 18

3 Abbreviations and Definitions

AE	Adverse Event
ASIA	American Spinal Injury Association
CT	Computed Tomography
EQ-5D	European Quality of life-5 Dimensions
MCS	Mental Component Summary
MRI	Magnetic Resonance Imaging
NPSI	Neuropathic Pain Symptom Inventory
OPLL	Ossification of the Posterior Longitudinal Ligament
PCS	Physical Component Summary
SAP	Statistical Analysis Plan
SCI	Spinal Cord Injury
SCIM III	Spinal Cord Independence Measure version III
SF-36	Short-Form Health Survey-36
WISCI II	Walking Index for Spinal Cord Injury II

4 Introduction

4.1 Preface

The optimal management of acute traumatic cervical spinal cord injury (SCI) associated with preexisting canal stenosis is unknown.

4.2 Scope of the analyses

These analyses will assess the efficacy and safety of early surgical decompression within 24 hours after admission for incomplete SCI without concomitant spinal fracture or dislocation, in comparison with delayed surgeries following at least two weeks of conservative treatment.

5 Study Objectives and Endpoints

5.1 Study Objectives

(ICH E3; 8)

The aim of this study is to test the hypothesis that early surgery (within 24 hours after admission) will lead to greater improvements in the motor function compared to delayed surgery (later than two weeks after injury) in patients with acute cervical SCI associated with canal stenosis. The aim of this study was to determine whether the early surgical decompression (<24h) results in better neurological recovery compared to the delayed surgery following conservative treatment for at least two weeks.

5.2 Endpoints

(ICH E9; 2.2.2)

Primary endpoints:
The primary outcome is a recovery in motor function one year after injury. The assessment will include:
1) the change from baseline to one year after the admission in the American Spinal Injury Association (ASIA) motor score;
2) the total score of the Spinal Cord Independence Measure version III (SCIM III);
3) the proportion of patients who regained the ability to walk 100 meters without human assistance.

The ASIA motor score is a 100-point score based on ten pairs of key muscles, each given a five point rating [1]. The SCIM III is a validated 100-point disability scale developed specifically for patients with SCI, with an emphasis on daily tasks grouped into three subscales: self-care (20 points), respiration and sphincter management (40 points) and mobility (40 points) [2].

Secondary endpoints:
The secondary outcomes will include:
1) the health-related quality of life as measured by the Medical Outcomes Study Short Form 36 (SF-36) [3] and the EuroQol 5 Dimension (EQ-5D) [4,5]
2) the neuropathic pain at the injured level and below as assessed by the Neuropathic Pain Symptom Inventory (NPSI) [6]
3) the walking status as evaluated with the Walking Index for Spinal Cord Injury (WISCI) II [7]

The scores on the SF-36 will be used as a generic measure of the patient health status. The NPSI is a self-questionnaire specifically designed to evaluate the different symptoms of neuropathic pain. It includes 12 items, each of which is quantified on a (0 to 10) numerical scale. The pain associated with SCI is classified into two categories: at-level pain and below-level pain. Participants will be asked to complete the NPSI separately for pain in the upper extremities (at-level pain) and in the trunk and lower extremities (below-level pain). The WISCI II is a valid 21-level hierarchical scale of walking based on physical assistance, the need for braces and devices, with an ordinal range from 0 (unable to walk) to 20 (walking without assistance for at least 10 meters).

6 Study Methods

6.1 General Study Design and Plan

(ICH E3; 9)

Study configuration and experimental design: randomized, parallel, controlled trial

Type of control(s): delayed surgery (a different treatment strategy)
Level and method of blinding: non-blinded. Physicians and research nurses who were not involved in the patient’s care assessed the outcome.

Method of treatment assignment: Randomization with stratification

At what point in time subjects are randomized relative to treatments, events and study periods：The subjects were randomized on their admission.

Sequence and duration of all study periods: described as below.

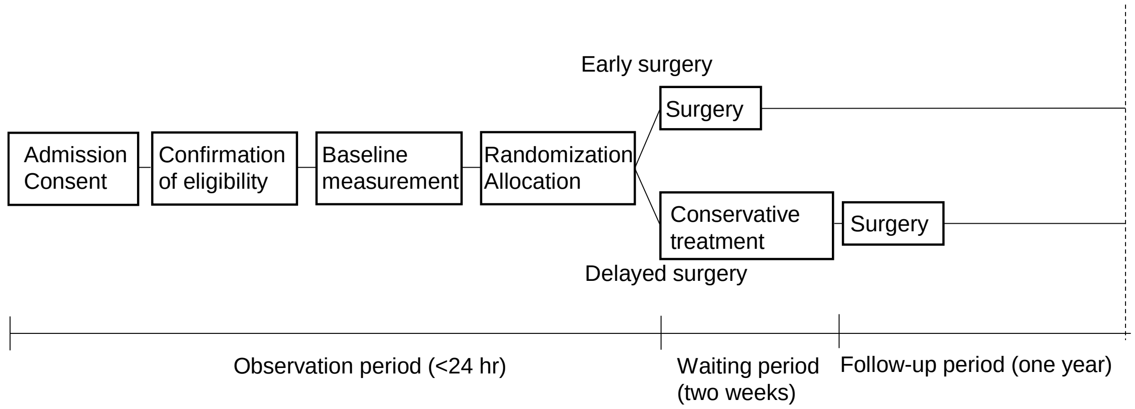

6.2 Equivalence or Non-Inferiority Studies

(ICH E3; 9.2, 9.7.1, 11.4.2.7. ICH E9; 3.3.2)

N/A

6.3 Inclusion-Exclusion Criteria and General Study Population

(ICH E3; 9.3. ICH E9; 2.2.1)

Inclusion Criteria:

- Acute traumatic cervical SCI
- 20 to 79 years of age
- ASIA Impairment Scale C
- Cervical canal stenosis confirmed based on findings of magnetic resonance imaging (MRI) and computed tomography (CT) due to preexisting conditions, such as spondylosis and ossification of the posterior longitudinal ligament without bone injury (spinal fracture or dislocation)

Exclusion Criteria:

- Unstable medical status
- Unable to undergo surgery within 24 hours after admission
- Impaired consciousness or mental disorder that precludes neurological examination

- Difficulty in obtaining informed consent

6.4 Randomization and Blinding

(ICH E3; 9.4.3, 9.4.6. ICH E9; 2.3.1, 2.3.2)

One to one randomization will be performed with the use of a Web-based system run by the University Medical Information Network that enabled computer-generated random treatment assignment. Randomization will be stratified according to the presence of Ossification of the Posterior Longitudinal Ligament (OPLL), the use of high-dose methylprednisolone treatment, the presence of preexisting gait disturbance due to myelopathy, and the presence severe canal compromise (>50%) by CT.

Physicians and research nurses who are not involved in the patient's care assess the outcome at each visit before seeing their doctors.

6.5 Study Variables

(ICH E3; 9.5.1. ICH E9; 2.2.2)

	Admission	2 week follow-up	3 month follow-up	6 month follow-up	1 year follow-up
Target day of visit		14	90	180	365
protocol assessment time windows (days)		+ 3	+ 14	+ 14	+ 14
Visit and examination	x	x	x	x	x
Baseline clinical characteristics	x				
Blood analyses	x	x	x	x	x
Magnetic resonance imaging	x				x

Computed tomography	x				
Plain radiographs	x	x			x
Neurological assessment including the ASIA motor score and ASIA impairment scale	x	x	x	x	x
SCIM III	x	x	x	x	x
SF-36	x				x
EQ-5D	x	x	x	x	x
NPSI	x				x
WISCI II	x				x

Variables	Description
ASIA motor score	range, 0 to 100, higher score indicating better motor recovery, based on ten pairs of key muscles, each given a five point
Spinal Cord Independence Measure (SCIM) version III	range, 0 to 100, higher score indicating better activity of daily living
SF-36	physical and mental component summary scores will be used.<br><br>range, 0 to 100, higher score indicating better status
EQ-5D	EQ-5D utility score will be used.<br><br>range, 0 to 1, higher score indicating better status

Neuropathic Pain Symptom Inventory (NPSI)	range, 0-100, higher score indicating more severe pain
Walking Index for Spinal Cord Injury (WISCI) II	range, 0 (unable to walk)-20 (walking without assistance for at least 10 meters, higher score indicating better walking status)

7 Sample Size

(ICH E3; 9.7.2. ICH E9; 3.5)

Sample size: 50 patients per group (total 100 patients)

For this exploratory trial, the sample size is determined primarily based on feasibility. We estimate that a sample of 45 patients per group would be sufficient to detect a difference of 12 points in the ASIA motor score when the standard deviation is 20. Additionally, assuming the percentage of ambulatory patients one year after the injury would increase from 50% to 80%, we need 39 patients for each group. Regarding the SCIM III, no data are available that can be used as a basis for sample size calculation. For the reasons above, we set the sample size to be 50 patients per group. All calculations assume an 80% power at a two-tailed significance level of 0.05.

8 General Considerations

8.1 Timing of Analyses

The final analysis will be performed when the last subjects have completed 1-year follow-up or dropped out prior to 1-year follow-up.

8.2 Analysis Populations

(ICH E3; 9.7.1, 11.4.2.5. ICH E9; 5.2)

8.2.1 Full Analysis Population

The full analysis population will consist of all randomized patients. The subjects who decline to participate before treatment will be excluded.

8.2.2 Per Protocol Population

N/A

8.2.3 Safety Population

All subjects who received any study treatment (including observation) but excluding subjects who drop out prior to receiving any treatment.

The primary and secondary analyses will be performed in full analysis population. Data for background characteristic will be collected from full analysis population. Data for safety will be collected from safety population.

8.3 Covariates and Subgroups

(ICH E3; 9.7.1, 11.4.2.1. ICH E9; 5.7)

The main analysis is an unadjusted analysis, even if there are factors that differ in patient background factors. If time-dependent covariates are found, they are evaluated at each time point only if the overall analysis is significant. However, since this is an exploratory analysis, no adjustment for multiplicity will be made.

Predefined subgroup analyses will be performed for the following factors: use of high-dose methylprednisolone treatment, the presence of OPLL, preexisting gait disturbance, severe canal compromise (> 50% canal compromise), and central cord syndrome (defined as the upper extremity ASIA motor score being at least 10 points less than the lower extremity motor score).

8.4 Missing Data

(ICH E3; 9.7.1, 11.4.2.2. ICH E9; 5.3. EMA Guideline on Missing Data in Confirmatory Clinical Trials)

Missing data will not be substituted in all variables.

8.5 Interim Analyses and Data Monitoring

(ICH E3; 9.7.1, 11.4.2.3. ICH E9; 4.1, FDA Feb 2010 "Guidance for Industry Adaptive Design Clinical Trials for Drugs and Biologics")

Interim analyses are not planned.

8.5.1 Purpose of Interim Analyses

N/A

8.5.2 Planned Schedule of Interim Analyses

N/A

8.5.3 Scope of Adaptations
N/A

8.5.4 Stopping Rules
N/A

8.5.5 Analysis Methods to Minimise Bias
N/A

8.5.6 Adjustment of Confidence Intervals and p-values
N/A

8.5.7 Interim Analysis for Sample Size Adjustment
N/A

8.5.8 Practical Measures to Minimise Bias
N/A

8.5.9 Documentation of Interim Analyses
N/A

8.6 Multi-center Studies
(ICH E3; 9.7.1, 11.4.2.4. ICH E9; 3.2)

In all the participating hospitals, the treatment for SCI is performed by board-certified spine specialists. As for the surgical procedure, the cervical decompression surgery is a well-established procedure. Therefore, we do not consider the difference between hospitals.

8.7 Multiple Testing
(ICH E3; 9.7.1, 11.4.2.5. ICH E9; 2.2.5)

It is an exploratory study and also aims to examine whether it can be evaluated for validation testing. Therefore, individual endpoints will be evaluated independently.

9 Summary of Study Data

All continuous variables will be summarised using the following descriptive statistics: n (non-missing sample size), mean, and 95% confidence interval. The frequency and percentages will be reported for all categorical measures.

All summary tables will be structured with a column for each treatment in the order (Early surgery, Delayed surgery) and will be annotated with the total population size relevant to that table.

9.1 Subject Disposition

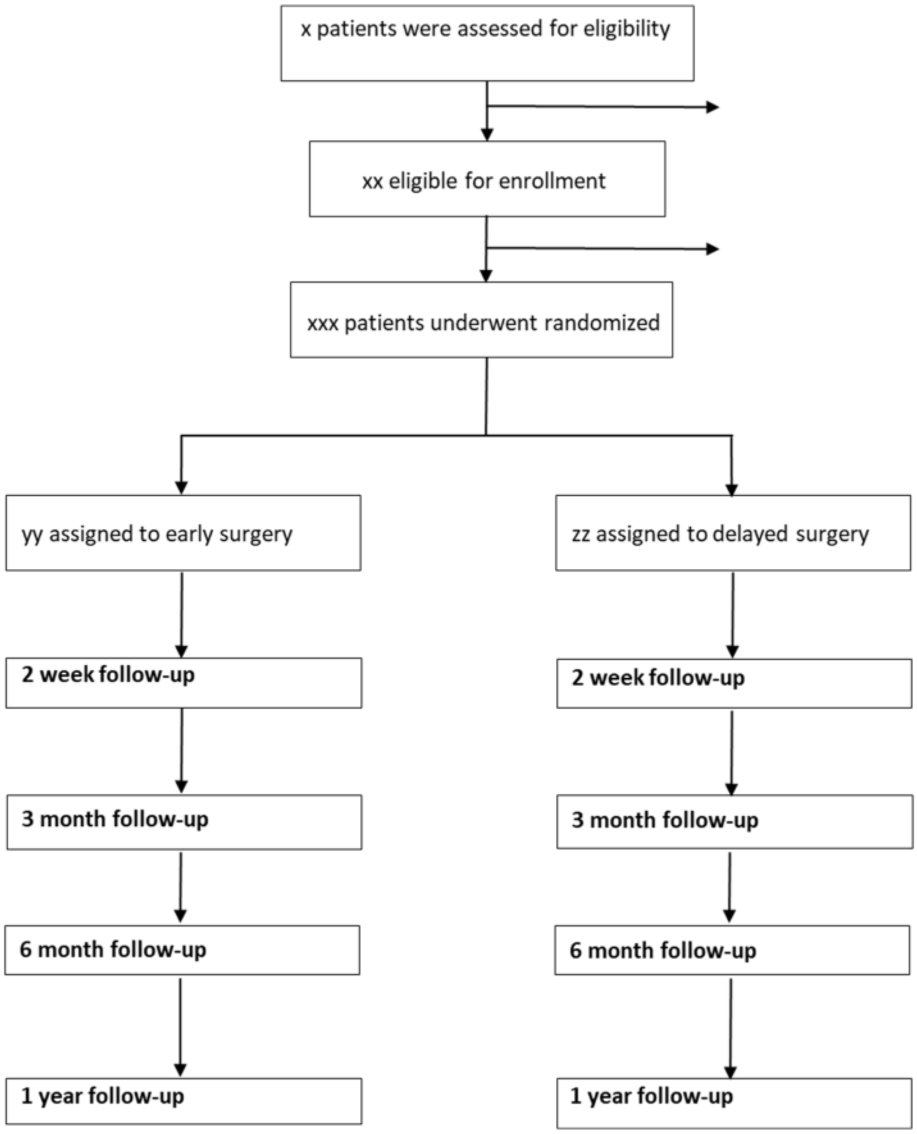

Consent withdraw cases will be removed at any time point.
Death cases will be included in analyses.

9.2 Derived variables

Physical component summary (PCS) and Mental component summary (MCS) scores of SF-36 will be calculated by using the methods set out by Fukuhara, Ware, et al. [3] and Fukuhara, Bito, et al [4].

EQ-5D utility score will be calculated from the raw score values using the value set for Japanese population (Tsuchiya A, Ikeda S, et al.) [5].

9.3 Protocol Deviations

Those who decline before treatment will be excluded.

9.4 Demographic and Baseline Variables

Age—yr

Male sex—no.(%)

Etiology—no.(%) ; Fall, Motor vehicle accident, Sports, Other

Time from injury to admission—median (IQR) (min)

OPLL—no.(%)

Occupancy rate > 50%—no.(%)

Preexisting gait disturbance due to myelopathy—no.(%)

Motor neurologic level of injury at admission—no.(%);

9.5 Concurrent Illnesses and Medical Conditions

N/A

9.6 Prior and Concurrent Medications

N/A

9.7 Treatment Compliance

N/A

10 Efficacy Analyses

All analyses of the continuous efficacy variables will be performed using repeated analysis of variance.

The proportion of patients will be compared using chi-square test.

The efficacy variables at single time point will be analysed using Student's t-test.

Treatment groups will be tested at the 2-sided 5% significance level.

10.1 Primary Efficacy Analysis

ASIA motor Score	The differences between the treatment groups will be compared using repeated analysis of variance. At the same time, the change from baseline to one year after the admission will be compared between the treatment groups using Student's t test.
The proportion of independent walkers	The proportion of patients who regained the ability to walk 100 meters without human assistance will be compared between the treatment groups using chi-square test.
SCIM Ⅲ	The differences between the treatment groups will be compared using repeated analysis of variance. At the same time, the total score of SCIM Ⅲ at one year after admission will be compared between the treatment groups using Student's t test.

10.2 Secondary Efficacy Analyses

SF-36	PCS and MCS scores at one year after the admission will be calculated and compared between the treatment groups using Student's t test.
EQ-5D	The utility scores of the EQ-5D at one year after the admission will be calculated and compared between the treatment groups using Student's t test.
Neuropathic Pain Symptom Inventory	The scores for pain at the injured level (arm) and below (trunk) will be separately assessed. Then the score at one year after the admission will be compared between the treatment groups using Student's t test.

WISCI II	The score of WISCI II (at two weeks and one year) will be compared between the treatment groups using Student's t test.

10.3 Exploratory Efficacy Analyses

N/A

11 Safety Analyses

The occurrence of prespecified adverse events will be assessed at each follow-up.

11.1 Extent of Exposure

The summary statistics will be produced in accordance with section 9.

11.2 Adverse Events

The a priori defined adverse events are: worsening of paralysis in the upper extremities, worsening of paralysis in the lower extremities, reoperation, use of a respirator (more than one week), tracheostomy, sepsis, pneumonia, acute respiratory distress syndrome, atelectasis, other respiratory complications, wound infection (superficial), wound infection (deep), urinary tract infection, other infections, gastrointestinal bleeding, peptic ulcer, ileus, acute myocardial infarction, other cardiac events, pulmonary embolism, cerebrovascular complication, liver dysfunction/disease, renal dysfunction/disease, delirium, depression, other complications and death.

When calculating the incidence of adverse events, the number of events will be counted, considering any repetitions of adverse events in the same patients; the denominator will be the total population size.

11.3 Deaths, Serious Adverse Events and other Significant Adverse Events

Severe AE: death, worsening of paralysis, unexpected hospital stay extension

11.4 Pregnancies

The summary statistics will be produced in accordance with section 9.

11.5 Clinical Laboratory Evaluations

The summary statistics will be produced in accordance with section 9.

11.6 Other Safety Measures

N/A

12 Pharmacokinetics

N/A

13 Other Analyses

N/A

14 Reporting Conventions

P-values ≥0.001 will be reported to 3 decimal places; P-values ≥0.01 will be reported to 2 decimal places; p-values less than 0.001 will be reported as "<0.001". The mean, standard deviation, and any other statistics other than quantiles, will be reported to one decimal place greater than the original data. Quantiles, such as median, or minimum and maximum will use the same number of decimal places as the original data.

15 Technical Details

Statistical analyses will be performed using JMP software.

The reviewing statistician will have an overview of the entire analyses.

16 References

1. American Spinal Injury Association. International standards for neurological classification of spinal cord injury (revised 2000). American Spinal Injury Association: Chicago, IL, 2002.
2. Itzkovich M, Gelernter I, Biering-Sorensen F, Weeks C, Laramee MT, Craven BC, Tonack M, Hitzig SL, Glaser E, Zeilig G, Aito S, Scivoletto G, Mecci M, Chadwick RJ, El Masry WS, Osman A, Glass CA, Silva P, Soni BM, Gardner BP, Savic G, Bergström EM, Bluvshtein V, Ronen J, Catz A. The Spinal Cord Independence Measure (SCIM) version III: reliability and validity in a multi-center international study. Disabil Rehabil. 2007 Dec 30;29(24):1926-1933.

3. Fukuhara S, Ware JE Jr, Kosinski M, Wada S, Gandek B. Psychometric and clinical tests of validity of the Japanese SF-36 Health Survey. J Clin Epidemiol. 1998 Nov;51(11):1045-1053.
4. Fukuhara S, Bito S, Green J, Hsiao A, Kurokawa K. Translation, adaptation, and validation of the SF-36 Health Survey for use in Japan. J Clin Epidemiol. 1998;51(11):1037-1044.
5. Tsuchiya A, Ikeda S, Ikegami N, Nishimura S, Sakai I, Fukuda T, Hamashima C, Hisashige A, Tamura M. Estimating an EQ-5D population value set: the case of Japan. Health Econ. 2002 Jun;11(4):341-353.
6. Bouhassira D, Attal N, Fermanian J, Alchaar H, Gautron M, Masquelier E, Rostaing S, Lanteri-Minet M, Collin E, Grisart J, Boureau F. Development and validation of the Neuropathic Pain Symptom Inventory. Pain. 2004 Apr;108(3):248-257.
7. Dittuno PL, Ditunno JF Jr. Walking index for spinal cord injury (WISCI II): scale revision. Spinal Cord. 2001 Dec;39(12):654-656. Erratum in: Spinal Cord. 2009 Apr;47(4):349.

Statistical Analysis Plan

TRIAL FULL TITLE	Optimal treatment for Spinal Cord Injury associated with cervical canal Stenosis Study (OSCIS Study)
REGISTRATION NUMBER	NCT01485458; UMIN000006780
SAP VERSION	Ver. 1.2
SAP VERSION DATE	9/9/2020
TRIAL STATISTICIAN	Hiroshi Ohtsu
TRIAL CHIEF INVESTIGATOR	Hirotaka Chikuda
SAP AUTHOR	Hiroshi Ohtsu

1 SAP Signatures

I give my approval for the attached SAP for the randomized controlled trial entitled OSICS, dated 9/9/2020.

Chief Investigator

Name: Hirotaka Chikuda

Signature: ____________________

Date: 9/9/2020

Statistician

Name: Hiroshi Ohtsu

Signature: ____________________

Date: 9/9/2020

2 Table of Contents

1 SAP Signatures 1
2 Table of Contents 2
3 Abbreviations and Definitions 5
4 Introduction 6
4.1 Preface 6
4.2 Scope of the analyses 6
5 Study Objectives and Endpoints 6
5.1 Study Objectives 6
5.2 Endpoints 6
6 Study Methods 7
6.1 General Study Design and Plan 7
6.2 Equivalence or Non-Inferiority Studies 8
6.3 Inclusion-Exclusion Criteria and General Study Population 8
6.4 Randomization and Blinding 9
6.5 Study Variables 9
7 Sample Size 11
8 General Considerations 11
8.1 Timing of Analyses 11
8.2 Analysis Populations 11
8.2.1 Full Analysis Population 11
8.2.2 Per Protocol Population 11
8.2.3 Safety Population 12
8.3 Covariates and Subgroups 12
8.4 Missing Data 12
8.5 Interim Analyses and Data Monitoring 12
8.5.1 Purpose of Interim Analyses 12

8.5.2 Planned Schedule of Interim Analyses 12
8.5.3 Scope of Adaptations 13
8.5.4 Stopping Rules 13
8.5.5 Analysis Methods to Minimise Bias 13
8.5.6 Adjustment of Confidence Intervals and p-values 13
8.5.7 Interim Analysis for Sample Size Adjustment 13
8.5.8 Practical Measures to Minimise Bias 13
8.5.9 Documentation of Interim Analyses 13
8.6 Multi-centre Studies 13
8.7 Multiple Testing 13
9 Summary of Study Data 13
9.1 Subject Disposition 14
9.2 Derived variables 14
9.3 Protocol Deviations 15
9.4 Demographic and Baseline Variables 15
9.5 Concurrent Illnesses and Medical Conditions 15
9.6 Prior and Concurrent Medications 15
9.7 Treatment Compliance 15
10 Efficacy Analyses 15
10.1 Primary Efficacy Analysis 16
10.2 Secondary Efficacy Analyses 16
10.3 Exploratory Efficacy Analyses 17
11 Safety Analyses 17
11.1 Extent of Exposure 17
11.2 Adverse Events 17
11.3 Deaths, Serious Adverse Events and other Significant Adverse Events 17
11.4 Pregnancies 17
11.5 Clinical Laboratory Evaluations 17

11.6 Other Safety Measures 17
12 Pharmacokinetics 18
13 Other Analyses 18
14 Reporting Conventions 18
15 Technical Details 18
16 References 18

3 Abbreviations and Definitions

AE	Adverse Event
ASIA	American Spinal Injury Association
CT	Computed Tomography
EQ-5D	European Quality of life-5 Dimensions
MCS	Mental Component Summary
MRI	Magnetic Resonance Imaging
NPSI	Neuropathic Pain Symptom Inventory
OPLL	Ossification of the Posterior Longitudinal Ligament
PCS	Physical Component Summary
SAP	Statistical Analysis Plan
SCI	Spinal Cord Injury
SCIM III	Spinal Cord Independence Measure version III
SF-36	Short-Form Health Survey-36
WISCI II	Walking Index for Spinal Cord Injury II

4 Introduction

4.1 Preface

The optimal management of acute traumatic cervical spinal cord injury (SCI) associated with preexisting canal stenosis is unknown.

4.2 Scope of the analyses

These analyses will assess the efficacy and safety of early surgical decompression within 24 hours after admission for incomplete SCI without concomitant spinal fracture or dislocation, in comparison with delayed surgeries following at least two weeks of conservative treatment.

5 Study Objectives and Endpoints

5.1 Study Objectives

(ICH E3; 8)

The aim of this study is to test the hypothesis that early surgery (within 24 hours after admission) will lead to greater improvements in the motor function compared to delayed surgery (later than two weeks after injury) in patients with acute cervical SCI associated with canal stenosis. The aim of this study was to determine whether the early surgical decompression (<24h) results in better neurological recovery compared to the delayed surgery following conservative treatment for at least two weeks.

5.2 Endpoints

(ICH E9; 2.2.2)

Primary endpoints:
The primary outcome is a recovery in motor function one year after injury. The assessment will include:
1) the change from baseline to one year after the admission in the American Spinal Injury Association (ASIA) motor score;
2) the total score of the Spinal Cord Independence Measure version III (SCIM III);
3) the proportion of patients who regained the ability to walk 100 meters without human assistance.

The ASIA motor score is a 100-point score based on ten pairs of key muscles, each given a five point rating [1]. The SCIM III is a validated 100-point disability scale developed specifically for patients with SCI, with an emphasis on daily tasks grouped into three subscales: self-care (20 points), respiration and sphincter management (40 points) and mobility (40 points) [2].

Secondary endpoints:
The secondary outcomes will include:
1) the health-related quality of life as measured by the Medical Outcomes Study Short Form 36 (SF-36) [3] and the EuroQol 5 Dimension (EQ-5D) [4,5]
2) the neuropathic pain at the injured level and below as assessed by the Neuropathic Pain Symptom Inventory (NPSI) [6]
3) the walking status as evaluated with the Walking Index for Spinal Cord Injury (WISCI) II [7]

The scores on the SF-36 will be used as a generic measure of the patient health status. The NPSI is a self-questionnaire specifically designed to evaluate the different symptoms of neuropathic pain. It includes 12 items, each of which is quantified on a (0 to 10) numerical scale. The pain associated with SCI is classified into two categories: at-level pain and below-level pain. Participants will be asked to complete the NPSI separately for pain in the upper extremities (at-level pain) and in the trunk and lower extremities (below-level pain). The WISCI II is a valid 21-level hierarchical scale of walking based on physical assistance, the need for braces and devices, with an ordinal range from 0 (unable to walk) to 20 (walking without assistance for at least 10 meters).

6 Study Methods

6.1 General Study Design and Plan

(ICH E3; 9)

Study configuration and experimental design: randomized, parallel, controlled trial

Type of control(s): delayed surgery (a different treatment strategy)
Level and method of blinding: non-blinded. Physicians and research nurses who were not involved in the patient's care assessed the outcome.

Method of treatment assignment: Randomization with stratification
At what point in time subjects are randomized relative to treatments, events and study periods：The subjects were randomized on their admission.
Sequence and duration of all study periods: described as below.

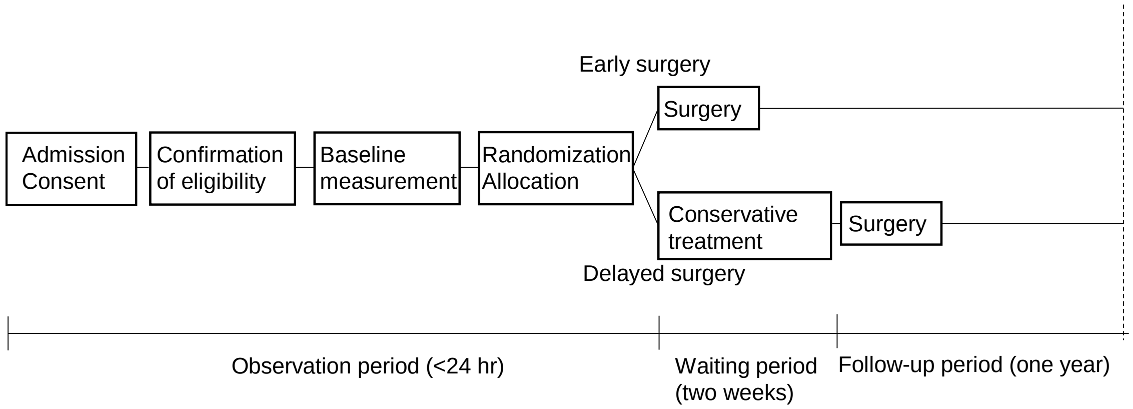

6.2 Equivalence or Non-Inferiority Studies

(ICH E3; 9.2, 9.7.1, 11.4.2.7. ICH E9; 3.3.2)

N/A

6.3 Inclusion-Exclusion Criteria and General Study Population

(ICH E3; 9.3. ICH E9; 2.2.1)

Inclusion Criteria:

- Acute traumatic cervical SCI
- 20 to 79 years of age
- ASIA Impairment Scale C
- Cervical canal stenosis confirmed based on findings of magnetic resonance imaging (MRI) and computed tomography (CT) due to preexisting conditions, such as spondylosis and ossification of the posterior longitudinal ligament without bone injury (spinal fracture or dislocation)

Exclusion Criteria:

- Unstable medical status
- Unable to undergo surgery within 24 hours after admission
- Impaired consciousness or mental disorder that precludes neurological examination

- Difficulty in obtaining informed consent

6.4 Randomization and Blinding

(ICH E3; 9.4.3, 9.4.6. ICH E9; 2.3.1, 2.3.2)

One to one randomization will be performed with the use of a Web-based system run by the University Medical Information Network that enabled computer-generated random treatment assignment. Randomization will be stratified according to the presence of Ossification of the Posterior Longitudinal Ligament (OPLL), the use of high-dose methylprednisolone treatment, the presence of preexisting gait disturbance due to myelopathy, and the presence severe canal compromise (>50%) by CT.

Physicians and research nurses who are not involved in the patient's care assess the outcome at each visit before seeing their doctors.

6.5 Study Variables

(ICH E3; 9.5.1. ICH E9; 2.2.2)

	Admission	2 week follow-up	3 month follow-up	6 month follow-up	1 year follow-up
Target day of visit		14	90	180	365
protocol assessment time windows (days)		+ 3	+ 14	+ 14	+ 14
Visit and examination	x	x	x	x	x
Baseline clinical characteristics	x				
Blood analyses	x	x	x	x	x
Magnetic resonance imaging	x				x

Computed tomography	x				
Plain radiographs	x	x			x
Neurological assessment including the ASIA motor score and ASIA impairment scale	x	x	x	x	x
SCIM III	x	x	x	x	x
SF-36	x				x
EQ-5D	x	x	x	x	x
NPSI	x				x
WISCI II	x				x

Variables	Description
ASIA motor score	range, 0 to 100, higher score indicating better motor recovery, based on ten pairs of key muscles, each given a five point
Spinal Cord Independence Measure (SCIM) version III	range, 0 to 100, higher score indicating better activity of daily living
SF-36	physical and mental component summary scores will be used.<br><br>range, 0 to 100, higher score indicating better status
EQ-5D	EQ-5D utility score will be used.<br><br>range, 0 to 1, higher score indicating better status

Neuropathic Pain Symptom Inventory (NPSI)	range, 0-100, higher score indicating more severe pain
Walking Index for Spinal Cord Injury (WISCI) II	range, 0 (unable to walk)-20 (walking without assistance for at least 10 meters, higher score indicating better walking status)

7 Sample Size

(ICH E3; 9.7.2. ICH E9; 3.5)

Sample size: 50 patients per group (total 100 patients)

For this exploratory trial, the sample size is determined primarily based on feasibility. We estimate that a sample of 45 patients per group would be sufficient to detect a difference of 12 points in the ASIA motor score when the standard deviation is 20. Additionally, assuming the percentage of ambulatory patients one year after the injury would increase from 50% to 80%, we need 39 patients for each group. Regarding the SCIM III, no data are available that can be used as a basis for sample size calculation. For the reasons above, we set the sample size to be 50 patients per group. All calculations assume an 80% power at a two-tailed significance level of 0.05.

8 General Considerations

8.1 Timing of Analyses

The final analysis will be performed when the last subjects have completed 1-year follow-up or dropped out prior to 1-year follow-up.

8.2 Analysis Populations

(ICH E3; 9.7.1, 11.4.2.5. ICH E9; 5.2)

8.2.1 Full Analysis Population

The full analysis population will consist of all randomized patients. The subjects who decline to participate before treatment will be excluded.

8.2.2 Per Protocol Population

N/A

8.2.3 Safety Population

All subjects who received any study treatment (including observation) but excluding subjects who drop out prior to receiving any treatment.

The primary and secondary analyses will be performed in full analysis population. Data for background characteristic will be collected from full analysis population. Data for safety will be collected from safety population.

8.3 Covariates and Subgroups

(ICH E3; 9.7.1, 11.4.2.1. ICH E9; 5.7)

The main analysis is an unadjusted analysis, even if there are factors that differ in patient background factors. If time-dependent covariates are found, they are evaluated at each time point only if the overall analysis is significant. However, since this is an exploratory analysis, no adjustment for multiplicity will be made.

Predefined subgroup analyses will be performed for the following factors: the presence of OPLL, severe canal compromise (> 50% canal compromise), and central cord syndrome (defined as the upper extremity ASIA motor score being at least 10 points less than the lower extremity motor score).

8.4 Missing Data

(ICH E3; 9.7.1, 11.4.2.2. ICH E9; 5.3. EMA Guideline on Missing Data in Confirmatory Clinical Trials)

Missing data will not be substituted in all variables.

8.5 Interim Analyses and Data Monitoring

(ICH E3; 9.7.1, 11.4.2.3. ICH E9; 4.1, FDA Feb 2010 "Guidance for Industry Adaptive Design Clinical Trials for Drugs and Biologics")

Interim analyses are not planned.

8.5.1 Purpose of Interim Analyses

N/A

8.5.2 Planned Schedule of Interim Analyses

N/A

8.5.3 Scope of Adaptations
N/A

8.5.4 Stopping Rules
N/A

8.5.5 Analysis Methods to Minimise Bias
N/A

8.5.6 Adjustment of Confidence Intervals and p-values
N/A

8.5.7 Interim Analysis for Sample Size Adjustment
N/A

8.5.8 Practical Measures to Minimise Bias
N/A

8.5.9 Documentation of Interim Analyses
N/A

8.6 Multi-center Studies
(ICH E3; 9.7.1, 11.4.2.4. ICH E9; 3.2)

In all the participating hospitals, the treatment for SCI is performed by board-certified spine specialists. As for the surgical procedure, the cervical decompression surgery is a well-established procedure. Therefore, we do not consider the difference between hospitals.

8.7 Multiple Testing
(ICH E3; 9.7.1, 11.4.2.5. ICH E9; 2.2.5)

It is an exploratory study and also aims to examine whether it can be evaluated for validation testing. Therefore, individual endpoints will be evaluated independently.

9 Summary of Study Data
All continuous variables will be summarised using the following descriptive statistics: n (non-missing sample size), mean, and 95% confidence interval. The frequency and percentages will be reported for all categorical measures.

All summary tables will be structured with a column for each treatment in the order (Early surgery, Delayed surgery) and will be annotated with the total population size relevant to that table.

9.1 Subject Disposition

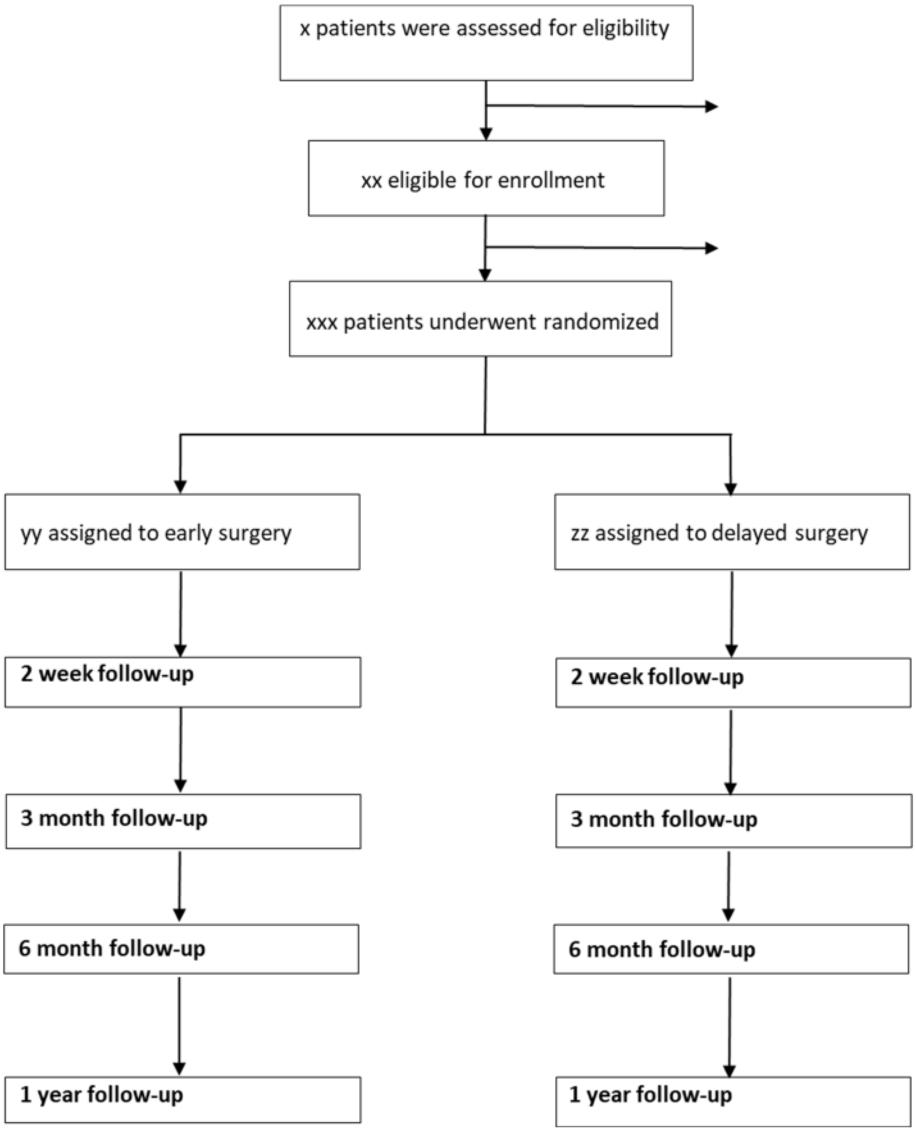

Consent withdraw cases will be removed at any time point.
Death cases will be included in analyses.

9.2 Derived variables

Physical component summary (PCS) and Mental component summary (MCS) scores of SF-36 will be calculated by using the methods set out by Fukuhara, Ware, et al. [3] and Fukuhara, Bito, et al [4].

EQ-5D utility score will be calculated from the raw score values using the value set for Japanese population (Tsuchiya A, Ikeda S, et al.) [5].

9.3 Protocol Deviations

Those who decline before treatment will be excluded.

9.4 Demographic and Baseline Variables

Age—yr

Male sex—no.(%)

Etiology—no.(%) ; Fall, Motor vehicle accident, Sports, Other

Time from injury to admission—median (IQR) (min)

OPLL—no.(%)

Occupancy rate > 50%—no.(%)

Preexisting gait disturbance due to myelopathy—no.(%)

Motor neurologic level of injury at admission—no.(%);

9.5 Concurrent Illnesses and Medical Conditions

N/A

9.6 Prior and Concurrent Medications

N/A

9.7 Treatment Compliance

N/A

10 Efficacy Analyses

All analyses of the continuous efficacy variables will be performed using repeated analysis of variance.

The proportion of patients will be compared using chi-square test.

The efficacy variables at single time point will be analysed using Student's t-test.

Treatment groups will be tested at the 2-sided 5% significance level.

10.1Primary Efficacy Analysis

ASIA motor Score	The differences between the treatment groups will be compared using repeated analysis of variance. At the same time, the change from baseline to one year after the admission will be compared between the treatment groups using Student's t test.
The proportion of independent walkers	The proportion of patients who regained the ability to walk 100 meters without human assistance will be compared between the treatment groups using chi-square test.
SCIM Ⅲ	The differences between the treatment groups will be compared using repeated analysis of variance. At the same time, the total score of SCIM Ⅲ at one year after admission will be compared between the treatment groups using Student's t test.

10.2 Secondary Efficacy Analyses

SF-36	PCS and MCS scores at one year after the admission will be calculated and compared between the treatment groups using Student's t test.
EQ-5D	The utility scores of the EQ-5D at one year after the admission will be calculated and compared between the treatment groups using Student's t test.
Neuropathic Pain Symptom Inventory	The scores for pain at the injured level (arm) and below (trunk) will be separately assessed. Then the score at one year after the admission will be compared between the treatment groups using Student's t test.
WISCI Ⅱ	The score of WISCI Ⅱ (at two weeks and one year) will be compared between the treatment groups using Student's t test.

10.3 Exploratory Efficacy Analyses

N/A

11 Safety Analyses

The occurrence of prespecified adverse events will be assessed at each follow-up.

11.1Extent of Exposure

The summary statistics will be produced in accordance with section 9.

11.2 Adverse Events

The a priori defined adverse events are: worsening of paralysis in the upper extremities, worsening of paralysis in the lower extremities, reoperation, use of a respirator (more than one week), tracheostomy, sepsis, pneumonia, acute respiratory distress syndrome, atelectasis, other respiratory complications, wound infection (superficial), wound infection (deep), urinary tract infection, other infections, gastrointestinal bleeding, peptic ulcer, ileus, acute myocardial infarction, other cardiac events, pulmonary embolism, cerebrovascular complication, liver dysfunction/disease, renal dysfunction/disease, delirium, depression, other complications and death.

When calculating the incidence of adverse events, the number of events will be counted, considering any repetitions of adverse events in the same patients; the denominator will be the total population size.

11.3 Deaths, Serious Adverse Events and other Significant Adverse Events

Severe AE: death, worsening of paralysis, unexpected hospital stay extension

11.4 Pregnancies

The summary statistics will be produced in accordance with section 9.

11.5 Clinical Laboratory Evaluations

The summary statistics will be produced in accordance with section 9.

11.6 Other Safety Measures

N/A

12 Pharmacokinetics

N/A

13 Other Analyses

N/A

14 Reporting Conventions

P-values ≥0.001 will be reported to 3 decimal places; P-values ≥0.01 will be reported to 2 decimal places; p-values less than 0.001 will be reported as "<0.001". The mean, standard deviation, and any other statistics other than quantiles, will be reported to one decimal place greater than the original data. Quantiles, such as median, or minimum and maximum will use the same number of decimal places as the original data.

15 Technical Details

Statistical analyses will be performed using JMP software.

The reviewing statistician will have an overview of the entire analyses.

16 References

1. American Spinal Injury Association. International standards for neurological classification of spinal cord injury (revised 2000). American Spinal Injury Association: Chicago, IL, 2002.
2. Itzkovich M, Gelernter I, Biering-Sorensen F, Weeks C, Laramee MT, Craven BC, Tonack M, Hitzig SL, Glaser E, Zeilig G, Aito S, Scivoletto G, Mecci M, Chadwick RJ, El Masry WS, Osman A, Glass CA, Silva P, Soni BM, Gardner BP, Savic G, Bergström EM, Bluvshtein V, Ronen J, Catz A. The Spinal Cord Independence Measure (SCIM) version III: reliability and validity in a multi-center international study. Disabil Rehabil. 2007 Dec 30;29(24):1926-1933.
3. Fukuhara S, Ware JE Jr, Kosinski M, Wada S, Gandek B. Psychometric and clinical tests of validity of the Japanese SF-36 Health Survey. J Clin Epidemiol. 1998 Nov;51(11):1045-1053.
4. Fukuhara S, Bito S, Green J, Hsiao A, Kurokawa K. Translation, adaptation, and validation of the SF-36 Health Survey for use in Japan. J Clin Epidemiol. 1998;51(11):1037-1044.

5. Tsuchiya A, Ikeda S, Ikegami N, Nishimura S, Sakai I, Fukuda T, Hamashima C, Hisashige A, Tamura M. Estimating an EQ-5D population value set: the case of Japan. Health Econ. 2002 Jun;11(4):341-353.
6. Bouhassira D, Attal N, Fermanian J, Alchaar H, Gautron M, Masquelier E, Rostaing S, Lanteri-Minet M, Collin E, Grisart J, Boureau F. Development and validation of the Neuropathic Pain Symptom Inventory. Pain. 2004 Apr;108(3):248-257.
7. Dittuno PL, Ditunno JF Jr. Walking index for spinal cord injury (WISCI II): scale revision. Spinal Cord. 2001 Dec;39(12):654-656. Erratum in: Spinal Cord. 2009 Apr;47(4):349.

Amendments to the Original Statistical Analysis Plan

Ver 1.2 September 9, 2020

No.	item	Before changed	After changed	Rationale
1	8-3. Covariates and Subgroups	Predefined subgroup analyses will be performed for the following factors: use of high-dose methylprednisolone treatment, the presence of OPLL, preexisting gait disturbance, severe canal compromise (> 50% canal compromise), and central cord syndrome (defined as the upper extremity ASIA motor score being at least 10 points less than the lower extremity motor score).	Predefined subgroup analyses will be performed for the following factors: the presence of OPLL, severe canal compromise (> 50% canal compromise), and central cord syndrome (defined as the upper extremity ASIA motor score being at least 10 points less than the lower extremity motor score).	Since number of subjects for the subgroup of methylprednisolone treatment and the subgroup of having preexisting gait disturbance were too few for the analysis, those subgroup analyses were excluded.

Abbreviations: ASIA, American Spinal Injury Association; OPLL, ossification of the posterior longitudinal ligament
