# Supplementary material for: Effect of Early vs Delayed Surgical Treatment on Motor Recovery in Incomplete Cervical Spinal Cord Injury With Preexisting Cervical Stenosis: A Randomized Clinical Trial
Source: JAMA Netw Open. 2021 Nov 9;4(11):e2133604. doi: 10.1001/jamanetworkopen.2021.33604 (PMC8579238; doi:10.1001/jamanetworkopen.2021.33604)
Supplement: Supplement 2. — eTable 1. Eligibility Criteria for Participants eTable 2. Intraoperative Adverse Events eTable 3. Baseline Characteristics of the Study Participants With or Without Primary Outcomes at 1 year eTable 4. Adverse Events and Complications During the Follow-up Period eFigure 1. Study Flowchart eFigure 2. Change in Motor Neurological Level of Injury During the Study Period, According to Treatment Group eFigure 3. Mean SCIM Score During the Study Period, According to Treatment Group eFigure 4. Mean EQ-5D Utility Score During the Study Period, According to Treatment Group [file jamanetwopen-e2133604-s002.pdf]

Supplemental Online Content

The OSCIS investigators. Effect of early vs delayed surgical treatment on motor recovery in incomplete cervical spinal cord injury with preexisting cervical stenosis: a randomized clinical trial. *JAMA Netw Open*. 2021;4(11):e2133604.
doi:10.1001/jamanetworkopen.2021.33604

eTable 1. Eligibility Criteria for Participants
eTable 2. Intraoperative Adverse Events
eTable 3. Baseline Characteristics of the Study Participants With or Without Primary Outcomes at 1 year
eTable 4. Adverse Events and Complications During the Follow-up Period
eFigure 1. Study Flowchart
eFigure 2. Change in Motor Neurological Level of Injury During the Study Period, According to Treatment Group
eFigure 3. Mean SCIM Score During the Study Period, According to Treatment Group
eFigure 4. Mean EQ-5D Utility Score During the Study Period, According to Treatment Group

This supplemental material has been provided by the authors to give readers additional information about their work.

© 2021 The OSCIS investigators. *JAMA Network Open.*

eTable 1. Eligibility Criteria for Participants

**Inclusion Criteria:**<br>● Aged 20 to 79 years<br>● ASIA Impairment Scale C[a]<br>● Cervical canal stenosis confirmed based on findings of magnetic resonance imaging and computed tomography due to pre-existing conditions, such as spondylosis and ossification of the posterior longitudinal ligament<br>● Without bone injury (spinal fracture or dislocation)
**Exclusion Criteria:**<br>● Unstable medical status<br>● Unable to undergo surgery within 24 hours after admission<br>● Impaired consciousness or mental disorder that precludes neurological examination<br>● Difficulty in obtaining informed consent

Abbreviation: ASIA, American Spinal Injury Association.

[a] Motor function is preserved below the neurological level, and more than half of key muscles below the neurological level have a muscle grade of < 3.

eTable 2. Intraoperative Adverse Events

	Early surgery (n=37)		Delayed surgery (n=26)*		P Value
Type of surgery					
Laminoplasty	34		24		0·95
Posterior decompression and fusion	3		2		
Time (min), median, (IQR)	133 (108-169)		150 (121-171)		0·36
Blood loss (mL), median, (IQR)	136 (44-250)		105 (60-214)		0·97
Nighttime surgery	10		1		
Allogeneic blood transfusion	4		0		
Stroke	1		0		
Worsening of paralysis	2		0		
Incidental durotomy	2		1		
Continuing intubation after surgery	10		2		

Abbreviation: IQR, interquartile range.

[a] Seven of 33 patients assigned to the delayed group did not undergo surgery within 1 year.

[b] P values were calculated using chi square test for "type of surgery" and Wilcoxon test for "time" and "blood loss."

[c] Nighttime surgery indicates operations that started between 8 pm and 6 am.

[d] Following prespecified adverse events did not occur in either treatment group: myocardial infarction, pulmonary embolism, vertebral artery injury, esophageal injury, pneumothorax, allergy, compressive peripheral nerve palsy, and wrong-level surgery.

© 2021 The OSCIS investigators. *JAMA Network Open.*

eTable 3. Baseline Characteristics of the Study Participants With or Without Primary Outcomes at 1 year

	**Primary Outcome at 1 year (N=56)**		**No Primary Outcome at 1 year[a] (N=14)**	**P-value**
Early surgery / Delayed surgery	30/26		7/7	0.81
Age—years	63.6±9.4		71.4±6.4	0.004
Male sex—no.(%)	51 (91)		14 (100)	0.25
OPLL—no.(%)	12 (32)		12 (36)	0.62
ASIA motor score at admission	33.0±12.4		33.7±12.6	0.86
Central cord syndrome—no.	16		6	0.57

Abbreviations: OPLL, ossification of the posterior longitudinal ligament; ASIA, American Spinal Injury Association.

[a] Including 6 patients who died during the study period.

[b] Values are expressed as mean ±SD.

[C] P values were calculated using chi square test except for “age” and “ASIA motor score” for which Student’s t–test was used.

© 2021 The OSCIS investigators. *JAMA Network Open.*

eTable 4. Adverse Events and Complications During the Follow-up Period

	**Early surgery (N=37)**		**Delayed surgery (N=33)**	
Death	3		3	
Worsening of paralysis （within 3 months）	3		4	
Worsening of paralysis （after 3 months）	3		2	
Reoperation	1		0	
Tracheostomy	2		2	
Sepsis	1		1	
Pneumonia	4		4	
Atelectasis	2		2	
Other respiratory complications	0		1 pleuritis	
Surgical site infection (superficial)	1		0	
Surgical site infection (deep)	0		1	
Urinary tract infection	12		7	
Other infection	2		1	
Gastrointestinal bleeding	0		1	
Gastric ulcer	0		1	
Other cardiac complications	0		1 arrhythmia	
Pulmonary embolism	1		0	
Liver disfunction	0		2	
Delirium	3		6	
Depression	1		4	
Other minor complications	12		16	

[a] This table included all serious adverse events that occurred before the 1-year follow-up, but were not necessarily caused by the treatment. The number of patients who developed complications is shown.

[b] Following prespecified adverse events did not occur in either treatment group: acute respiratory distress syndrome, ileus, acute coronary syndrome, cerebrovascular complications, or renal dysfunction.

© 2021 The OSCIS investigators. *JAMA Network Open.*

eFigure 1. Study Flowchart

eFigure 1. Study flow

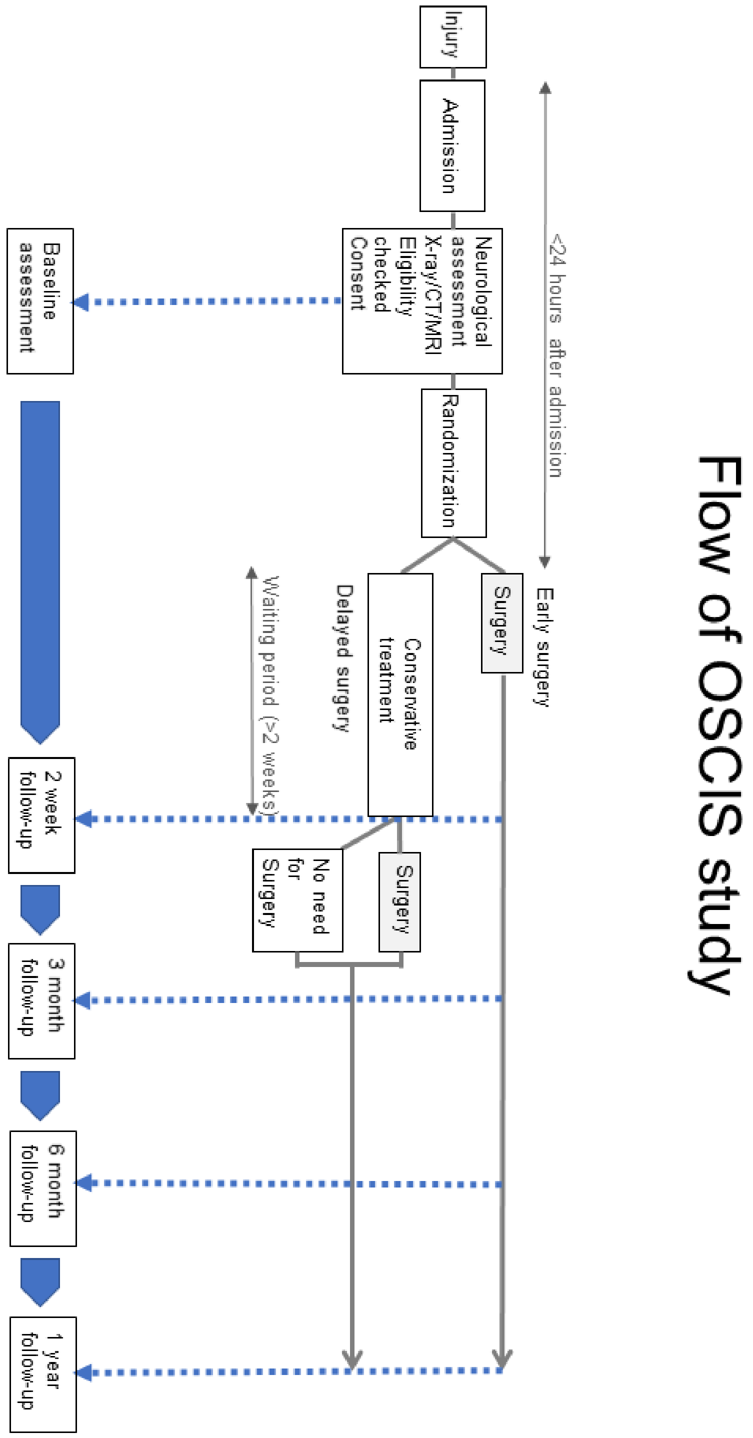

© 2021 The OSCIS investigators. *JAMA Network Open.*

eFigure 2. Change in Motor Neurological Level of Injury During the Study Period, According to Treatment Group

eFigure 2. Change in Motor Neurological Level of Injury during the Study Period, According to Treatment Group

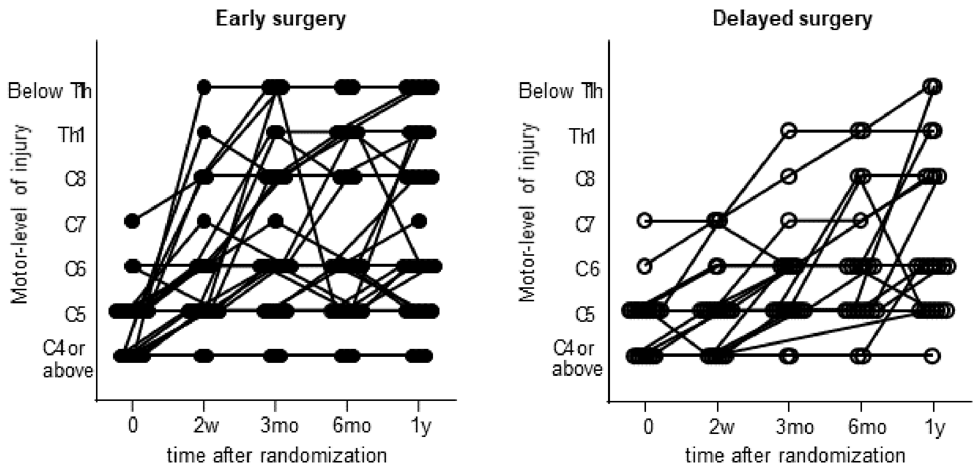

The graph shows the most caudal segment of the spinal cord with antigravity motor function on both sides of each case. When the functional level reaches lower limbs, the case is described as "Below Th1".

© 2021 The OSCIS investigators. *JAMA Network Open.*

eFigure 3. Mean SCIM Score During the Study Period, According to Treatment Group

eFigure 3. Mean SCIM Score during the Study Period, According to Treatment Group

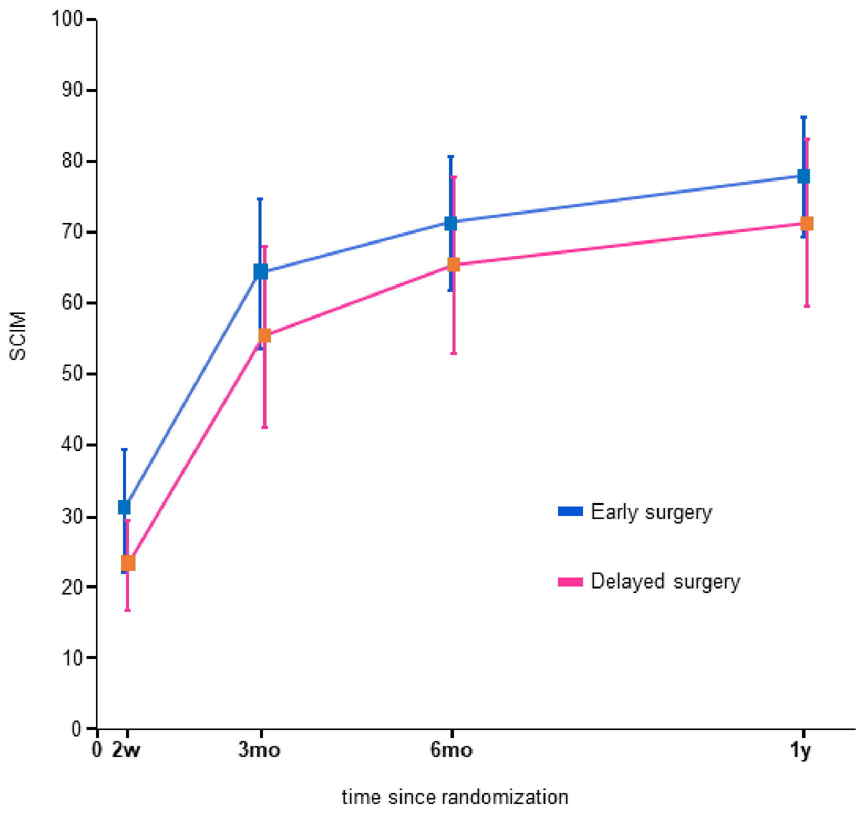

No. of Patients	0 w	2w	3mo	6mo	1y
Early surgery	X	37	35	33	30
Delayed surgery	X	33	27	24	23

The graph shows the mean total Spinal Cord Independence Measure (SCIM) score for groups randomly assigned to early surgery within 24 hours after admission or to receive delayed surgery following at least 2 weeks of conservative treatment. The I bars indicate 95% confidence intervals.

© 2021 The OSCIS investigators. *JAMA Network Open.*

eFigure 4. Mean EQ-5D Utility Score During the Study Period, According to Treatment Group

eFigure 4. Mean EQ-5D Utility Score during the Study Period, According to Treatment Group

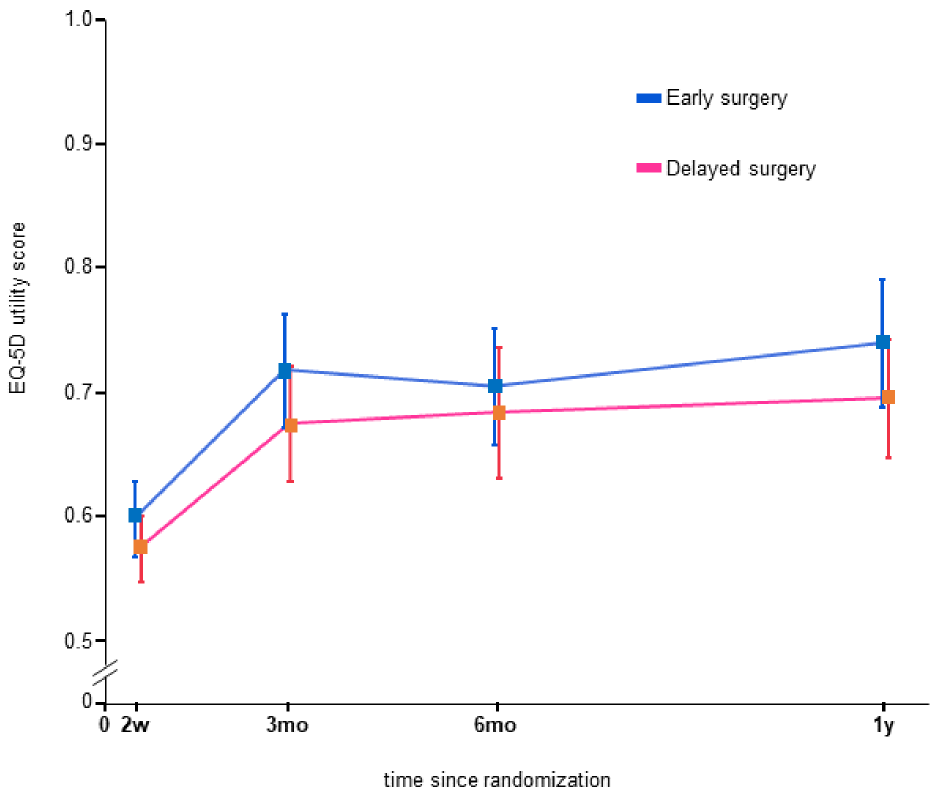

No. of Patients	0 w	2w	3mo	6mo	1y
Early surgery	X	37	33	32	28
Delayed surgery	X	32	25	20	22

The graph shows the mean European Quality of Life-5 Dimension (EQ-5D) utility score for groups randomly assigned to early surgery within 24 hours after admission or to receive delayed surgery following at least 2 weeks of conservative treatment. The I bars indicate 95% confidence intervals.

© 2021 The OSCIS investigators. *JAMA Network Open.*
