# Supplementary material for: Effect of Early vs Delayed Surgical Treatment on Motor Recovery in Incomplete Cervical Spinal Cord Injury With Preexisting Cervical Stenosis: A Randomized Clinical Trial
Source: JAMA Netw Open. 2021 Nov 9;4(11):e2133604. doi: 10.1001/jamanetworkopen.2021.33604 (PMC8579238; doi:10.1001/jamanetworkopen.2021.33604)
Supplement: Supplement 3. — Data Sharing Statement [file jamanetwopen-e2133604-s003.pdf]

Data Sharing Statement

Chikuda. Effect of Early vs Delayed Surgical Treatment on Motor Recovery in Incomplete Cervical Spinal Cord Injury With Preexisting Cervical Stenosis. *JAMA Netw Open*. Published November 09, 2021. doi:10.1001/jamanetworkopen.2021.33604

Data
Data available: Yes
Data types: Deidentified participant data
How to access data: chikuda-tky@umin.ac.jp
When available: beginning date: 12-01-2023

Supporting Documents
Document types: None

Additional Information
Who can access the data: researchers whose proposed use of the data has been approved
Types of analyses: for a specified purpose
Mechanisms of data availability: after approval of a proposal
